# The pangenome: a statistical model, not a fixed biological property

**DOI:** 10.1093/bioadv/vbag069

**Published:** 2026-04-01

**Authors:** Abraham Guerra

**Affiliations:** Research Network for Open and Independent Science (RICAI), 080001, Colombia

## Abstract

The pangenome, representing the complete genetic repertoire of a species, has become a central concept in modern prokaryotic genomics, moving the field beyond the limitations of a single reference genome. This review provides a comprehensive overview of the pangenomic landscape, from its conceptual foundations to its diverse applications. We critically examine the methodological and statistical underpinnings of pangenome analysis, emphasizing that the pangenome is not a fixed biological entity but a statistical model whose outputs are fundamentally dependent on dataset quality, curation, and taxonomic resolution. We discuss the transition from the simple open/closed dichotomy to more nuanced, ecologically driven models like pangenome fluidity and the ecogenome, which better explain the evolutionary dynamics of prokaryotic genomes. With a special focus on the archaeal pangenome, we highlight the unique challenges and novel evolutionary insights emerging from this under-explored domain. As the field moves towards graph-based representations, AI-driven analysis, and the expansion into metapangenomics integrated with functional multi-omics, we argue that a rigorous and critical approach to data interpretation will be paramount to unlocking a true understanding of microbial evolution and function.

## 1 Introduction

Over the past two decades, there has been an exponential increase in the amount of available genomic data, primarily driven by the advent and refinement of next-generation sequencing technologies. These innovations have led to drastic reductions in sequencing costs, improvements in data acquisition speed, and increased accuracy of results (Shendure and Ji 2008, Land *et al.* 2015). This trend has often been compared to Moore’s Law, which describes the exponential growth in computational processing power, a pattern that has similarly manifested in genomics, particularly in the dramatic reduction of the cost per billion base pairs sequenced (Mueller 2020, Bornstein *et al.* 2023).

**Figure 1:**
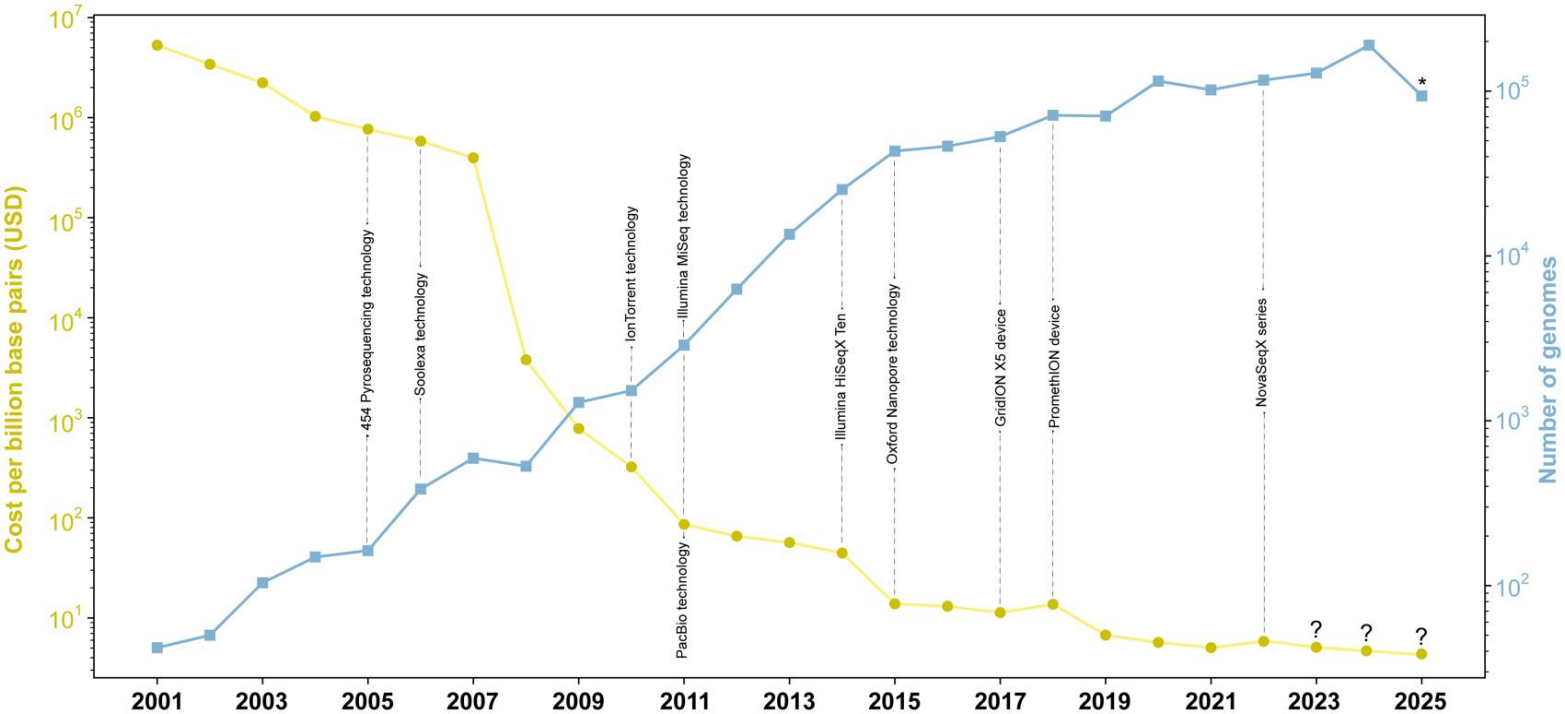
Schematic representation comparing the cost per billion base pairs (USD) and the number of genomes submitted in the NCBI database from 2001 to 2025. The cost trend is depicted in yellow, while the cumulative number of genomes is shown in blue. Both axes use logarithmic scales to facilitate data comparison. The “?” symbol indicates projected data or ideal trends where current data are unavailable, while “*” denotes that the values for 2025 are incomplete, but the expected trend is an ongoing increase. Key milestones in sequencing technology development are highlighted at specific years. https://www.genome.gov/about-genomics/fact-sheets/DNA-Sequencing-Costs-Data and https://ourworldindata.org/grapher/cost-of-sequencing-a-full-human-genome

The magnitude of this transformation becomes evident when comparing sequencing costs: in 2001, sequencing one billion base pairs exceeded USD 5 million, whereas today the cost is approximately USD 5 (Fig. 1). This remarkable decrease has paralleled the development and implementation of key sequencing technologies, such as sequencing by synthesis, Single Molecule Real-Time sequencing, and nanopore sequencing, pioneered by Illumina (2006), Pacific Biosciences (2011), and Oxford Nanopore Technologies (2014), respectively.

This cost reduction and the broad dissemination of these technologies have enabled not only the large-scale sequencing of cultivable organisms but also of those that were previously inaccessible. In fact, it is estimated that only between 0.1% and 1% of prokaryotic species are cultivable under standard laboratory conditions (Amann *et al.* 1995, Colwell 1997, Tyson and Banfield 2005, Li *et al.* 2023, Simon *et al.* 2025).

To overcome these cultivation barriers, culture-independent approaches such as metagenomics emerged, allowing the recovery of genetic information directly from complex microbial communities, without the need for isolation or cultivation. This approach targets what is often referred to as the “microbial dark matter” or the “uncultivated majority” (Rinke *et al.* 2013, Zha *et al.* 2022, Li *et al.* 2023).

A pivotal milestone in this context was the publication of the first metagenome-assembled genomes (MAGs) in 2004 (Tyson *et al.* 2004). MAGs are reconstructed genomes obtained directly from environmental metagenomic sequencing data, without the need of cultivation (Hugerth *et al.* 2015, Parks *et al.* 2017, Setubal 2021, Li *et al.* 2023). Since then, thousands of MAGs have been sequenced greatly expanding our understanding of genetic diversity across a wide range of environments, including soils, aquatic ecosystems, plant microbiomes, the human body, and extreme habitats such as volcanoes, hydrothermal vents, and hypersaline zones (Albertsen *et al.* 2013, Hugerth *et al.* 2015, Anantharaman *et al.* 2016, Parks *et al.* 2017, Chen *et al.* 2021, Chibani *et al.* 2022, Su *et al.* 2022, Zhang and Jing 2024). However, MAGs pose several challenges: the potential formation of chimeric assemblies, the inherent diversity of microbial communities, contamination, gene absence due to incomplete assemblies, and methodological differences in genome assembly and binning algorithms (Chen *et al.* 2020, Meziti *et al.* 2021). These factors can compromise both the quality and interpretability of genomes reconstructed through metagenomic approaches.

The development and massive availability of genomes have paved the way for comparative genomics, a field that enables the analysis of multiple genomes across different taxa to identify similarities and differences in their structures, evolutionary trajectories, gene content, and functional capacities (de Crécy-Lagard and Hanson 2013, Kawashima 2019, Bornstein *et al.* 2023). From this framework emerged pangenomics—a discipline that systematically studies the genetic diversity within a taxon. This approach is particularly relevant in prokaryotes, where even strains of the same species, isolated from the same environment at the same time, can display substantial genomic differences.

In this review, we provide a comprehensive overview of the field of prokaryotic pangenomics, from its conceptual origins to its modern applications. We place a special emphasis on the critical methodological and statistical considerations that underpin pangenome analysis, arguing that the pangenome must be understood not as a fixed biological entity but as a statistical model dependent on data curation. We explore the evolution of key concepts such as pangenome openness and fluidity, discuss challenges posed by metagenome-assembled genomes (MAGs), and highlight the unique insights emerging from the under-explored domain of Archaea.

## 2 The genesis of the pangenome concept

Historically, bacterial populations were perceived as arrays of stable lineages (clones) that maintained their chromosomal gene composition with little or no rearrangement overtime (Ørskov and Ørskov 1977, Selander and Levin 1980, Porras *et al.* 1986a, 1986b). Under this view, bacteria of identical genotypes could be isolated from distant geographical locations or at different times, suggesting a rigid population structure. *Escherichia coli* serving as a cornerstone model in microbiology, genetics, and evolution, was central to these early frameworks. Prior to the genomic era, our understanding of its diversity relied heavily on methods like Multilocus Enzyme Electrophoresis (MLEE) (Selander and Levin 1980, Caugant *et al.* 1981). These early studies hypothesized that *E. coli* populations were predominantly clonal, composed of a modest number of widespread and stable lineages with low rates of chromosomal recombination. They also noted that these populations harbored extensive genetic diversity—approximately twice that previously estimated (Milkman 1973) and 3× to 5× greater than typically recorded for eukaryotes (Milkman 1973, Selander and Levin 1980).

Subsequent work began to reveal a more dynamic picture. Studies on single-host populations demonstrated that while resident clones could persist, diversity was frequently driven by the invasion of new genotypes and the rapid turnover of plasmids, suggesting that chromosomal recombination played a minor role in generating this specific diversity (Caugant *et al.* 1981). These findings hinted at the crucial role of extrachromosomal elements in adaptation—conferring traits such as antibiotic resistance and atypical carbon fermentation—and suggested that significant genetic flexibility existed beyond the stable chromosome (Stewart and Levin 1977, Levin and Stewart 1980, Caugant *et al.* 1981). Concurrently, evidence of horizontal gene transfer (HGT) shaping bacterial genomes emerged (Médigue *et al.* 1991). The advent of Multilocus Sequence Typing (MLST) further refined this resolution by analyzing DNA sequences directly, uncovering even greater diversity within these clonal lineages (Maiden *et al.* 1998). By the late 1990s, the magnitude of this microbial world was becoming apparent; seminal estimates suggested that prokaryotes comprise the bulk of Earth’s biomass (4–6 × 10^3^ cells), with a staggering but largely unquantified species richness (Whitman *et al.* 1998, Gaston 2000).

This historical tension between a stable clonal backbone and a vast, dynamic accessory pool laid the conceptual groundwork for the pangenome. The limitations of the “single reference” paradigm finally became undeniable with the first multi-strain genome comparisons. A landmark study compared three *E. coli* strains (MG1655, O157: H7, and CFT073), revealing that they shared only about 39% of their genes (Welch *et al.* 2002). This “pangenome-like” observation hinted at a massive, unexplored gene pool and set the stage for the formal conceptualization of the pangenome. This tension between a stable clonal backbone and a dynamic accessory pool laid the conceptual groundwork for what would later be defined as the pangenome.

This recognition of extensive genomic plasticity soon collided with the practical challenges of the 21st century, particularly in infectious disease management. A striking example was the high incidence of life-threatening neonatal infections caused by Group B Streptococcus (GBS) (Pizza *et al.* 2000, Tettelin *et al.* 2000). A pioneering solution emerged through reverse vaccinology, a strategy that leveraged the first available bacterial genome sequences to predict surface-exposed proteins as potential vaccine candidates (Pizza *et al.* 2000, Tettelin *et al.* 2000). While revolutionary, this approach was initially based on a limited number of genomes, raising a critical question: how representative is a single genome of an entire species?

Early comparative genomic studies quickly revealed the limitations of a single-reference view. For instance, microarray-based comparative genomic hybridization (CGH) between GBS and other streptococci highlighted substantial genomic diversity and revealed that a significant fraction of genes was variable, even among isolates of the same serotype (Glaser *et al.* 2002, Tettelin *et al.* 2002). This accumulating evidence underscored that the genomic content of a single isolate was insufficient to capture the full genetic repertoire of a species.

This realization culminated in a landmark 2005 study that formally introduced the “pangenome” concept. By sequencing and analyzing eight GBS isolates, representing the five major disease-causing serotypes, researchers provided a stunning quantitative picture of genomic diversity (Tettelin *et al.* 2005). They discovered that only 80% of the genes constituted a “core genome” shared by all isolates, while the remaining 20% formed a “dispensable genome.” This seminal work established that the pangenome—composed of core and dispensable genes—was vast and that no two isolates were genetically identical.

The transition of pangenomics from a novel concept to a cornerstone of modern microbiology was fueled by a revolution in sequencing technology. The precipitous drop in sequencing costs, coupled with an exponential increase in the number of publicly available genomes, democratized the field and enabled large-scale comparative analyses that were previously inconceivable (Fig. 1).

Consequently, pangenome analyses have been applied to countless species across the tree of life. *Escherichia coli*, with its immense genomic plasticity and clinical relevance, has become a model organism for pangenomic research, providing deep insights into the mechanisms driving microbial evolution, adaptation, and niche specialization (Yang and Gao 2022, Chauhan *et al.* 2025). The principles uncovered in these early studies have since laid the groundwork for the entire field.

## 3 Defining the pangenome

**Figure 2:**
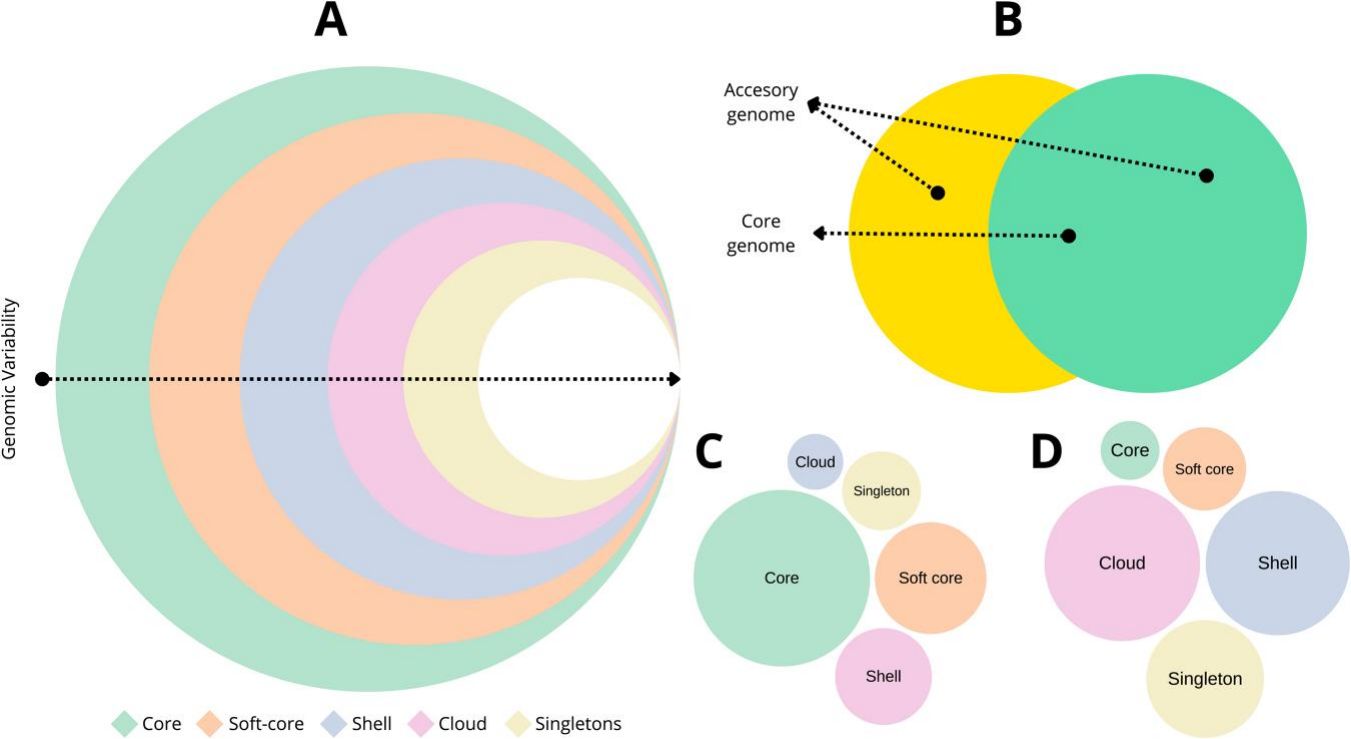
Composition of the pangenome. (A) Concentric-circle diagram showing different components, from universally conserved core genes to increasingly variable components: soft-core, shell, cloud, and singletons. Note that this is a conceptual representation; the relative size of each fraction varies significantly between taxa. (B) Venn diagram showing core genome and the accessory genome. (C) Model of a pangenome dominated by a large core genome, represented by five circles of decreasing size: core, soft-core, shell, singletons, and cloud. (D) Model of a pangenome with a reduced core and expanded accessory components, particularly the cloud and shell genes, ordered from largest to smallest: cloud, shell, singletons, soft-core, and core.

The pangenome represents the entire genetic repertoire of a given taxonomic unit, such as a species, encompassing all genes found across multiple genomes. It is fundamentally composed of two main parts: the core genome, comprising genes shared by all individuals, and the accessory genome, which includes all non-core genes (Fig. 2) (Tettelin and Medini 2020, Kaur *et al.* 2024, Matthews *et al.* 2024). The accessory genome is highly variable and can be further subdivided into strain-specific genes (or singletons), found in only one genome, and genes shared by a subset of genomes. Although pangenomic studies have been traditionally conducted at the species level, the approach is inherently flexible and can be applied at various taxonomic resolutions—ranging from genera and families to broader ecological scales such as entire communities, or even at finer scales like single-cell populations within a tissue (Matthews *et al.* 2024).

However, extending pangenome analysis to higher taxonomic ranks (e.g. genus or family) introduces significant methodological challenges, particularly in defining orthologous groups and necessitates a critical recalibration of gene clustering thresholds. As evolutionary distance increases, nucleotide sequence identity drops and synteny is disrupted, complicating the identification of orthologs (Rodriguez-R and Konstantinidis 2014, Sarton-Lohéac *et al.* 2025). Consequently, applying strict sequence identity cutoffs (e.g. ≥95%), which are standard for species-level analysis, to broader taxa often results in an artificial contraction of the core genome and an inflation of the accessory fraction. This occurs because orthologous genes often diverge beyond these fixed thresholds due to synonymous mutations, leading algorithms to misclassify shared homologs as distinct “singleton” or accessory genes. As demonstrated by Rodriguez-R and Konstantinidis (2014), nucleotide-based metrics like Average Nucleotide Identity (ANI) lose resolution below ∼80% identity, a boundary frequently crossed at the genus level (Rodriguez-R and Konstantinidis 2014). Therefore, to capture the true functional core at higher ranks, analyses must shift towards Amino Acid Identity (AAI) or protein structural domains. This dependency further underscores the central thesis of this review: the “size” and “nature” of a pangenome are not absolute biological constants, but statistical outputs heavily contingent upon the interplay between taxonomic resolution and bioinformatic parameters.

In parallel with these considerations, it is crucial to distinguish the pangenome from the ecogenome. While a pangenome is traditionally delimited by taxonomic boundaries (i.e. the gene repertoire of a specific taxon, such as a species) (Tettelin and Medini 2020), the ecogenome is defined by ecology (Smillie *et al.* 2011, Cordero and Polz 2014). It represents the set of genes shared by organisms occupying the same ecological niche, regardless of their evolutionary relationship. For example, distantly related bacteria in the human gut share an “intestinal ecogenome” comprising genes for specific carbohydrate degradation or bile resistance, acquired through horizontal gene transfer to survive in that shared environment (Smillie *et al.* 2011, Cordero and Polz 2014). Understanding this distinction prevents the conflation of taxonomic history with ecological adaptation when analyzing complex communities (Domingo-Sananes and McInerney 2021).

The classification of genes within the pangenome is, however, not uniform and can vary depending on methodological approaches and computational tools used. A widely adopted classification scheme, often referred to as gene frequency partitioning, subdivides gene categories based on their occurrence across genomes: core genes are those present in at least 99% of genomes; soft-core genes occur in 95% to 99%; shell genes are present in 15% to 95%; and cloud genes appear in less than 15% of genomes (Fig. 2) (Koonin and Wolf 2008). These thresholds, while commonly applied, are not absolute and can be adjusted according to the specific characteristics of the dataset, the evolutionary history of the taxon under study, or the particular objectives of the research. Importantly, such classifications are sensitive to parameter choices and may be influenced by technical artifacts inherent to genome assembly and annotation processes (Matthews *et al.* 2024).

The core genome generally includes genes associated with fundamental cellular functions such as replication, transcription, translation, and essential metabolic pathways (Tettelin *et al.* 2005, 2008, Yang and Gao 2022). However, core genes are not always synonymous with essential genes. Some essential genes might not be shared universally due to HGT, gene loss, or lineage-specific adaptations. Conversely, the accessory genome encompasses genes frequently associated with adaptive traits, including antibiotic resistance, virulence factors, environmental stress responses, and metabolic versatility (Tettelin *et al.* 2005, 2008, Yang and Gao 2022).The acquisition and loss of these genes often occur through mechanisms such as HGT, mobilization by genetic elements, or phage-mediated gene exchange (Tettelin *et al.* 2008, Yang and Gao 2022). Core genes are typically highly conserved and subjected to strong purifying selection, whereas accessory genes are more variable, often exhibiting relaxed selective constraints (Matthews *et al.* 2024). A further notable component of the pangenome is constituted by singleton genes (Fig. 2), which are unique to a single genome. These may result from recent gene acquisitions, genome decay, or, alternatively, may arise as artifacts from genome assembly and annotation processes (Chávez-Luzanía *et al.* 2022).

The relative sizes of each pangenome component can vary considerably among taxa. Some pangenomes exhibit a large core and soft-core with comparatively small shell, cloud, and singleton fractions, indicative of a highly conserved genomic feature (Fig. 2C). Conversely, other taxa display expansive shell and cloud components but a reduced core, reflecting higher genomic plasticity (Fig. 2D). These compositional differences offer insights into the evolutionary dynamics and ecological strategies of different organisms.

These concepts are powerfully illustrated by comparing organisms from different ecological contexts. For instance, endosymbiotic bacteria like *Candidatus Liberibacter asiaticus*, which inhabit stable intracellular environments, possess highly conserved, “closed” pangenomes with large core components (Darolt *et al.* 2021). Conversely, environmentally ubiquitous genera with complex lifestyles, such as *Chromobacterium* and *Collimonas*, exhibit remarkably “open” pangenomes with small core genomes and vast accessory components, reflecting extensive genomic plasticity and ecological adaptability (Gaba *et al.* 2020, Dimitrova *et al.* 2023).

Historically, pangenomes were analyzed using a presence-absence variation (PAV) model, which catalogs genes in a matrix format (Tettelin *et al.* 2005). While foundational, this approach struggles with the scale and complexity of modern datasets. In response, more sophisticated frameworks have emerged. One is the representative sequence model, which reduces redundancy by creating a curated, non-redundant set of sequences that captures maximal population-level diversity (Matthews *et al.* 2024).

The most transformative advance, however, is the pangenome graph. This model represents the pangenome as a network of nodes and edges, moving beyond linear references. Current approaches can be broadly categorized into gene-based graphs, which model gene adjacencies and functional modules (e.g. PPanGGOLiN, Panaroo), and sequence-based graphs, capable of encoding not just gene presence-absence but also complex structural variations, rearrangements, and SNPs (Gautreau *et al.* 2020, Tonkin-Hill *et al.* 2020). Pangenome graphs are rapidly becoming the new standard, offering a far more comprehensive and accurate view of genomic variation than traditional linear references (Eizenga *et al.* 2020).

The pangenome provides a comprehensive and dynamic perspective of genomic variation, transcending the limitations imposed by reliance on a single linear reference genome, while the relative proportions of core and accessory genomes offer key insights into population genetics: a reduced core genome typically reflects extensive genetic diversity, whereas an expanded core suggests a more conserved and genetically uniform population (Carlos Guimaraes *et al.* 2015, Matthews *et al.* 2024).

## 4 The pangenome as a statistical approximation of the total gene Pool

Accessing the full extent of genetic diversity within a taxon is currently unfeasible, as sequencing every strain or individual remains logistically impractical (Tettelin and Medini 2020, Matthews *et al.* 2024). Consequently, any computed pangenome is not an absolute biological truth but rather a statistical approximation of a taxon’s total genetic repertoire. The robustness of any pangenomic inference—from the core genome size to its open or closed nature—is therefore critically dependent on the size, quality, and representativeness of the input genomes. A biased or low-quality dataset can significantly skew the estimation of core and accessory components, leading to flawed biological conclusions. Therefore, while it is essential to include genomes from diverse ecological and geographical origins to capture maximal diversity, this inclusion must be governed by rigorous quality control standards (Li and Yin 2022).

## 5 Navigating the landscape of genomic data: from complete genomes to MAGs

**Figure 3:**
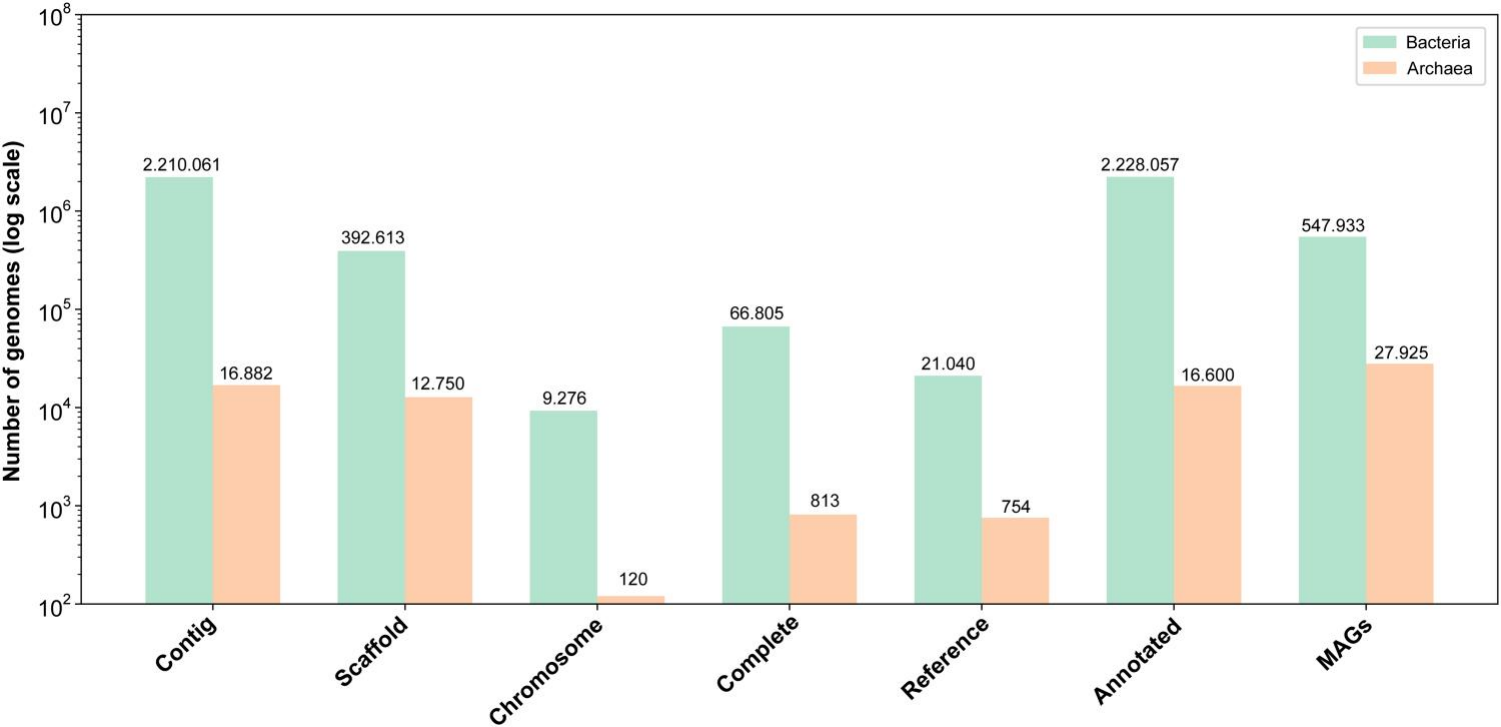
Bar chart comparing the number of prokaryotic genomes deposited in the NCBI database. Bacterial genomes are represented by green bars, while archaeal genomes are shown in orange, categorized by assembly level: Contig, Scaffold, Chromosome, Complete, Reference, Annotated, and MAGs. The *y*-axis uses a logarithmic scale to accommodate the large differences in genome counts, enhancing interpretability. The absolute number of genomes is indicated above each bar.

The genomic data available in public repositories is dominated by drafts of varying quality, rather than complete, high-quality isolates. Our analysis of over 3.2 million prokaryotic genomes in NCBI reveals the prevalence of different assembly levels (Fig. 3). A significant portion are MAGs; while they constitute a substantial fraction of bacterial genomes, this proportion becomes overwhelming in Archaea, where they represent a striking 91% of the total.

The reliance on MAGs stems from the dual challenge of accessing microbial diversity: most prokaryotes remain unculturable under standard laboratory conditions (Hugerth *et al.* 2015, Parks *et al.* 2017, Li *et al.* 2023), and even culture-independent surveys have significant detection biases (Brown *et al.* 2015). For instance, due to primer mismatches with divergent 16S rRNA gene sequences, it is estimated that 50%–100% of organisms in certain phyla can evade detection entirely (Brown *et al.* 2015). Overcoming these hurdles to recover complete genomes directly from environmental samples requires a sequencing depth and coverage that is often impractical or cost-prohibitive. As a result, MAGs have become an indispensable tool. Despite being frequently fragmented, incomplete, or contaminated, they represent our primary window into this diversity, a reality that underscores the necessity of stringent quality assessment before their inclusion in any pangenomic analysis (Li and Yin 2022).

The challenge of data quality is not exclusive to MAGs. An examination of all prokaryotic genomes by assembly level shows that the vast majority are highly fragmented. Approximately 82% of genomes are at the “Contig” level, followed by 14.9% at the “Scaffold” level. In contrast, a mere ∼2.86% are classified as “Complete” or “Chromosome” level assemblies. Critically, only about 0.8% of all genomes are designated as “Reference genomes,” suggesting that even the most reliable data represents a tiny fraction of the available genomes (Fig. 3). This emphasizes the need for caution when using genomes at lower assembly levels, as they may introduce inaccuracies similar to those associated with MAGs.

## 6 Essential quality metrics: completeness and contamination

Although greater genomic completeness yields more comprehensive and useful data (Chen *et al.* 2020), the central goal of pangenomics is to capture maximum genetic diversity within a given taxon. Given that the vast majority of genomes in public repositories are incomplete drafts—primarily MAGs and contig-level assemblies—achieving broad taxonomic representation requires their inclusion. This reality necessitates a core methodological principle: incorporating diverse, incomplete genomes is not only acceptable but essential for modern pangenomic studies, provided that each genome first passes stringent, standardized quality thresholds to ensure the reliability and integrity of the analysis.

Two primary metrics for genome quality assessment are completeness and contamination, routinely evaluated using tools such as CheckM, CheckM2, MAGISTA, BUSCO, and GUNC (Parks *et al.* 2015, Simão *et al.* 2015, Orakov *et al.* 2021, Goussarov *et al.* 2022, Chklovski *et al.* 2023). Completeness refers to the proportion of expected genes present in a genome based on conserved marker gene sets (Parks *et al.* 2015, Eisenhofer *et al.* 2023). Contamination, on the other hand, relates to the presence of unrelated genomic fragments, which can distort biological interpretations by introducing foreign genes and potentially leading to erroneous conclusions about the genome’s functional repertoire or evolutionary history (Orakov *et al.* 2021).

Contamination manifests in two principal forms. The first, redundant contamination, consists of excess genomic fragments from closely related organisms, often within the same lineage (Orakov *et al.* 2021). The second, non-redundant contamination, involves foreign genomic material from distantly related organisms resulting in chimeric assemblies (Orakov *et al.* 2021). Redundant contamination typically consists of additional copies of sequences already present within the genome, whereas non-redundant contamination introduces entirely novel sequences, making it more difficult to distinguish authentic genomic elements.

Accurately identifying contamination becomes especially problematic when dealing with novel or poorly characterized lineages for which no close reference genomes exist (Orakov *et al.* 2021). Given these risks, the inclusion of any genome—especially incomplete assemblies or MAGs—must be preceded by stringent quality control to safeguard the integrity of pangenome analyses.

## 7 Dataset curation: balancing diversity, representativeness, and size

A foundational principle in pangenome construction is that the representativeness and diversity of the dataset are more critical than the sheer number of genomes included (Yang and Gao 2022, Matthews *et al.* 2024) The inclusion of many phylogenetically redundant or highly similar genomes can severely bias the analysis. Specifically, over-sampling certain clades artificially inflates the core genome size while underestimating the accessory genome, which can lead to critical misinterpretations, such as misclassifying unique genes and wrongly characterizing a pangenome’s open or closed nature (Steinke *et al.* 2021, Wu *et al.* 2021, 2022, Carpi *et al.* 2022, Yang and Gao 2022).

Incorporating closely related strains (e.g. hundreds of isolates from a single clonal outbreak) introduces redundancy that artificially inflates the core genome size while masking the true openness of the pangenome (Steinke *et al.* 2021, Wu *et al.* 2021, 2022, Carpi *et al.* 2022, Yang and Gao 2022). To mitigate this, dereplication is a crucial step. Tools like dRep facilitate the removal of redundant genomes based on Average Nucleotide Identity (ANI) thresholds (typically ≥99% or ≥99.5% for strain-level redundancy) (Ondov *et al.* 2016, Olm *et al.* 2017). This process often relies on robust alignment algorithms such as FastANI, PYANI, or OrthoANI (Lee *et al.* 2016, Pritchard *et al.* 2016, Jain *et al.* 2018). Proper dereplication balances the dataset, preventing the overestimation of conserved genes and ensuring that pangenome metrics—such as fluidity and openness—accurately reflect the biological diversity of the taxon rather than sampling bias.

Furthermore, accurate taxonomic classification is a prerequisite for meaningful pangenomic comparisons. The NCBI Taxonomy database serves as the vital, comprehensive repository for nomenclature and organism discovery (Schoch *et al.* 2020, Cox *et al.* 2025). Building upon this foundational resource, the Genome Taxonomy Database (GTDB) refines these classifications to provide a standardized, genome-informed taxonomy. By utilizing Relative Evolutionary Divergence (RED) to normalize higher taxonomic ranks and Average Nucleotide Identity (ANI) to delineate species clusters, GTDB ensures that taxa at the same rank represent comparable evolutionary distances (Parks *et al.* 2022, 2026). Adopting this standardized framework minimizes the inclusion of misclassified genomes, thereby reducing noise in core and accessory genome estimates and facilitating more robust comparative analyses.

The question of “how many genomes are enough?” has no universal answer, as the ideal number is dictated by the intrinsic nature of the taxon’s pangenome. The nature of the pangenome–whether it is open or closed—directly impacts how many genomes should be included. Species with “open” pangenomes, often associated with ecologically diverse or sympatric lifestyles with high rates of HGT, require a larger and more varied set of genomes to begin capturing their full genetic repertoire (Rouli *et al.* 2015). Conversely, species with “closed” pangenomes, such as those that are intracellular or ecologically isolated (allopatric), may be adequately represented by a much smaller number of genomes (Rouli *et al.* 2015). Thus, effective dataset design requires a thoughtful strategy guided by the taxon’s ecological context and evolutionary history, rather than an arbitrary numerical cutoff.

## 8 Should pseudogenes be included in prokaryotic pangenome analyses?

Whether pseudogenes should be included in pangenomic analyses remains controversial. While their inclusion may introduce noise by incorporating non-functional gene remnants as if they were functional genes, conversely, excluding them entirely could erase evolutionary signals that inform gene loss dynamics, genome reduction, and adaptation strategies.

Pseudogenes are typically defined as genomic sequences that resemble functional genes but have lost their ability to encode functional proteins due to the accumulation of disabling mutations such as frameshifts, premature stop codons, insertions, or deletions (Smillie *et al.* 2011, Danneels *et al.* 2018). In prokaryotic genomes, pseudogenization may occur through neutral processes like genetic drift, especially when selective pressures to maintain gene functionality are relaxed, or, conversely, through adaptive processes aimed at eliminating deleterious gene products (Kuo *et al.* 2009, Smillie *et al.* 2011, Danneels *et al.* 2018, Douglas and Shapiro 2024).

Although microbial genomes are typically compact and characterized by high coding density, pseudogenes do exist and can arise either as natural consequences of evolutionary gene degradation or as technical artifacts introduced during genome sequencing, assembly, or annotation (Cooley and Wright 2024). Importantly, genomes with a substantial proportion of pseudogenes can compromise pangenomic analyses by inflating the apparent gene content or by misleading functional inference, particularly when pseudogenes are incorrectly annotated as protein-coding sequences (Yang and Gao 2022). Conversely, some pseudogenes may be entirely missed during annotation, further complicating assessments of gene presence or absence.

Interestingly, while pseudogenes tend to accumulate in certain genomes—especially those of host-associated or recently evolved bacteria—they are typically rare in free-living prokaryotes, where non-functional DNA tends to be rapidly purged to maintain genome streamlining and efficiency (Kuo and Ochman 2010, Danneels *et al.* 2018). This trend suggests that the presence of pseudogenes may be, deleterious, prompting their removal through purifying selection (Kuo and Ochman 2010).

Moreover, a recent study has shown that in prokaryotic pangenomes, when a genome contains a single functional copy of an accessory gene, it is often depleted of non-functional pseudogene variants from the same functional category (Nature Ecology and Evolution 2024). This observation supports an adaptive model of pangenome evolution, wherein the retention of functional genes is favored over their inactivated forms (Nature Ecology and Evolution 2024). Nevertheless, the functional role of pseudogenes in prokaryotes is generally negligible since they are typically not expressed or, if transcribed, do not produce functional proteins (Vanin 1985). Consequently, while they offer valuable insights into evolutionary trajectories, their inclusion in functional pangenomic analyses aimed at identifying active metabolic or adaptive capabilities may be misleading.

The decision to include or exclude pseudogenes in pangenomic analyses should be guided by the specific goals of the study. When the focus is related to evolutionary questions, their retention can reveal patterns of gene decay and adaptation. However, for functional characterization, careful curation is necessary to avoid conflating non-functional remnants with genuine coding sequences. This is particularly important given that many pseudogenes can be misannotated as coding sequences or overlooked altogether depending on the annotation pipeline used (Yang and Gao 2022). Therefore, while pseudogenes are an inherent component of prokaryotic genomes and hold significant evolutionary information, their role in pangenomic analyses must be carefully considered to avoid biases in functional inference and comparative approaches.

## 9 The open/closed dichotomy: a statistical model, not an intrinsic biological trait size

A central concept in pangenomics is the classification of a taxon’s pangenome as either “open” or “closed.” This describes how the total gene repertoire changes as more genomes are sampled. An open pangenome continuously expands with the addition of new genomes, while a closed pangenome contains a finite number of genes that is captured after sampling only a few individuals (Tettelin and Medini 2020). This behavior is most commonly quantified using Heaps’ law, an empirical power-law model expressed as *P = kN^γ^*, where *P* is the pangenome size, *N* is the number of genomes, *κ* is a fitting parameter, and the exponent *γ* (gamma) indicates the rate of new gene discovery (Tettelin *et al.* 2008).

Initially, the interpretation was a simple dichotomy: a pangenome was considered “open” if *γ* >  0 and “closed” if *γ* = 0 (Tettelin *et al.* 2008, Tettelin and Medini 2020). However, this binary view is overly simplistic, as virtually no pangenome is absolutely closed (*γ* = 0) due to the inevitable accumulation of rare mutations or transient horizontal gene transfer events. A more flexible and widely adopted approach classifies pangenomes on a continuum, for instance, using a threshold where *γ* < 0.3 suggests a closed nature and *γ* > 0.3 indicates an intermediate-to-open nature (Rajput *et al.* 2023). This framework better reflects the biological reality that openness is a spectrum, not a binary state.

Open pangenomes imply a high rate of gene turnover, frequent gene gain/loss events, and substantial genomic plasticity (Soares *et al.* 2013, Puigbò *et al.* 2014, Jaiswal *et al.* 2020, Domingo-Sananes and McInerney 2021, Cummins *et al.* 2022, Karampatakis *et al.* 2024). In such cases, even after sampling dozens or hundreds of strains, novel genes continue to emerge with nearly every genome added (Matthews *et al.* 2024). Conversely, in closed pangenomes, the species’ genetic repertoire is comprehensively sampled by a limited number of representatives, after which the addition of new genomes results in minimal or no gene discovery (Rouli *et al.* 2015).

**Figure 4:**
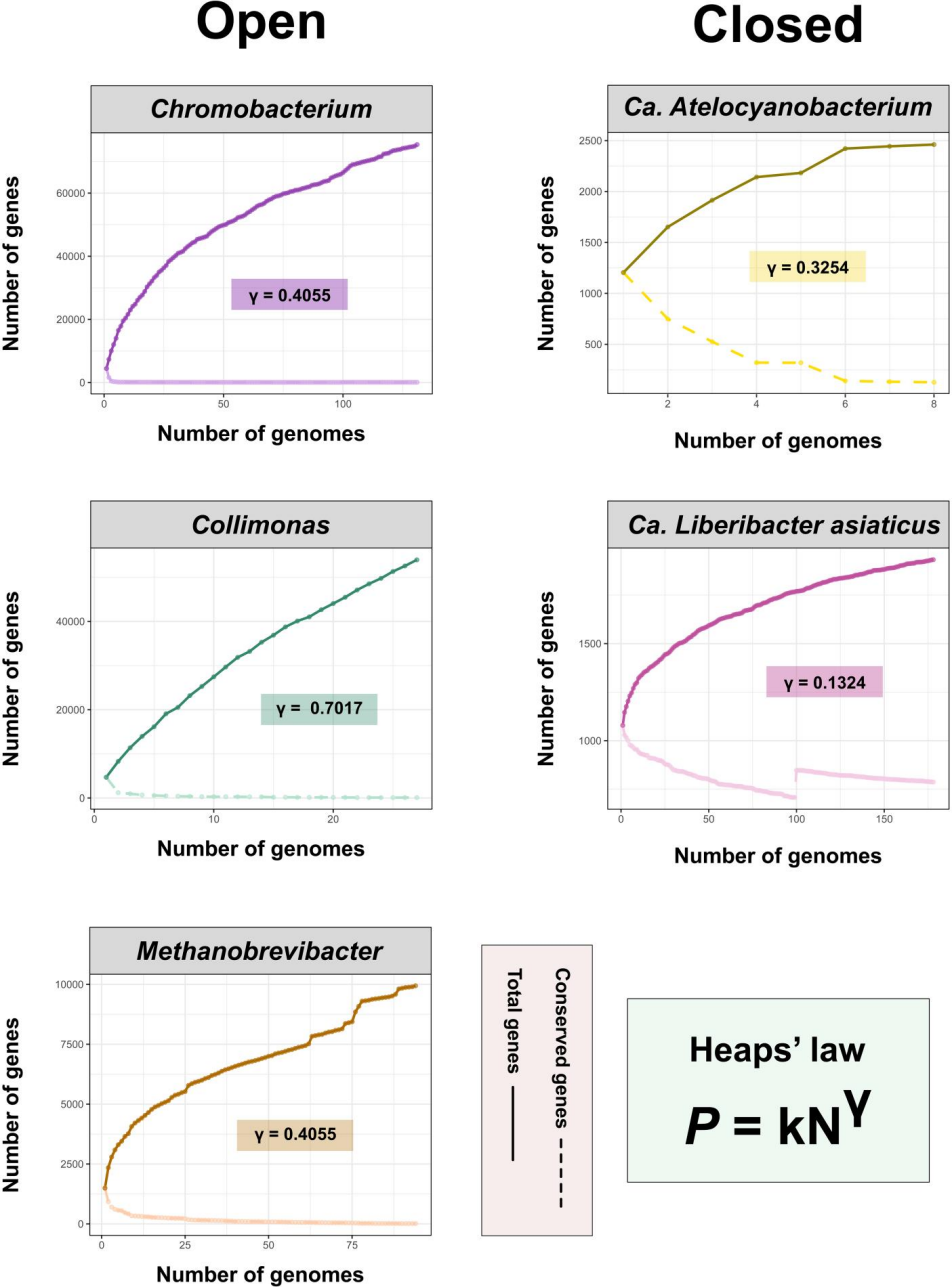
Comparative pangenome analysis of five prokaryotic taxa with different lifestyles. The figure displays the pangenome growth curve (total genes, solid line) and the core genome decay curve (conserved genes, dashed line) for the genera *Chromobacterium*, *Collimonas*, *Methanobrevibacter* (*M. smithii* and *M. intestinalis*), and *Candidatus Atelocyanobacterium*, and for the species *Candidatus Liberibacter asiaticus*. In each panel, the *x*-axis represents the number of genomes added sequentially, and the *y*-axis represents the number of genes. The openness of each pangenome was modeled using Heaps’ law (*P* = kN^γ^). Values of γ < 0.3 suggest a closed pangenome, while values of γ > 0.3 indicate an intermediate open pangenome.

This classification of pangenomes into open and closed types is clearly exemplified by our analysis of five distinct prokaryotic taxa (Fig. 4). The endosymbionts *Candidatus Liberibacter asiaticus* (*γ* = 0.132) and *Candidatus Atelocyanobacterium* (*γ* = 0.325) display closed pangenomes, consistent with their stable, isolated niches. In contrast, the environmentally ubiquitous genera *Chromobacterium* (*γ* = 0.465), *Collimonas* (*γ* = 0.702), and the gut-associated *Methanobrevibacter smithii/intestini* (*γ* = 0.407) all exhibit clearly open pangenomes, reflecting their extensive genomic plasticity and exposure to HGT.

Determining how many genomes are necessary to “define” a species’ pangenome is also not straightforward. While it is frequently assumed that large genome sets are required to capture species diversity, some taxa exhibit closed pangenomes with surprisingly few genomes. In *Bacillus anthracis*, for instance, a near-complete pangenome can be obtained from as strains due to its highly clonal population structure and low HGT rates (Zwick *et al.* 2012, Rouli *et al.* 2014, 2015, Zorigt *et al.* 2024) Similarly, *Staphylococcus lugdunensis* and *Lactobacillus acidophilus* show closed pangenomes despite low genome counts (Lebeurre *et al.* 2019, Rajput *et al.* 2023). In contrast, *E. coli*, *Legionella pneumophila*, and *Limosilactobacillus reuteri* exhibit open pangenomes, reflecting extensive gene flux and ecological adaptability (D’Auria *et al.* 2010, Tantoso *et al.* 2022, Zhang *et al.* 2024).

Critically, the “openness” or “closedness” of a pangenome should not be interpreted as an intrinsic biological truth but rather as an emergent property of the dataset analyzed—an artifact shaped by sampling depth, population structure, and annotation consistency. A systematic evaluation of 27 bacterial species, for example, found that in cases where the core/pangenome ratio remained above 98%, only two genomes were sufficient to approximate the entire gene repertoire (Rouli *et al.* 2015). For closed pangenomes, six genomes were often enough. In contrast, for open pangenomes, gene discovery remained active beyond ten genomes—and in some cases, failed to converge even with dozens of representatives (Rouli *et al.* 2015). These observations emphasize that dataset design and taxonomic resolution are critical in interpreting pangenomic dynamics. Sampling too few genomes may lead to incorrect inferences about pangenome structure. Conversely, over-sampling a group that has not been well-defined taxonomically (i.e. by mixing several closely related but distinct lineages) can produce an artificially open signature. The apparent nature of a pangenome is thus not fixed, but relative to how—and how well—we define the group under study.

## 10 Beyond open/closed: pangenome fluidity and the ecological drivers of genome evolution

The evolution of prokaryotic pangenomes is a dynamic process governed by factors that extend far beyond a simple open-or-closed classification. This dynamic nature is not an intrinsic, random property but is profoundly shaped by the ecological context and lifestyle of the species. In fact, species can be viewed on an ecological spectrum, from specialists in stable, isolated niches (allopatric) to generalists interacting within diverse communities (sympatric) (Rouli *et al.* 2015). To properly capture this complexity, it is essential to move beyond the open/closed dichotomy and embrace quantitative metrics such as genome fluidity (Kislyuk *et al.* 2011), proposed to better measure gene content diversity independent of the number of sequenced genomes (Table 1).

**Table 1:** Summary of key analytical frameworks and statistical models describing prokaryotic pangenomes.

Analytical framework/model	Description	Biological determinants	Methodological determinants
Presence-absence variation (PAV)	The foundational matrix representation cataloging gene content (0/1) across genomes. Basis for most accumulation curves.	Gene gain/loss events Horizontal Gene Transfer (HGT)	Annotation quality: gene prediction errors. Pseudogenes: may inflate accessory counts if not filtered.
Gene frequency partitioning	Classification of genes into discrete categories (Core, Soft-core, Shell, Cloud) based on their frequency of occurrence.	Selection pressures: strong purifying selection (Core) versus relaxed selective constraints (Accessory)	Taxonomic rank and thresholds: strict DNA thresholds at higher ranks artificially deflate the Core. Assembly artifacts: fragmentation and contamination misclassify genes.
Pangenome nature (openness)	Empirical power-law model (*P* = kN^γ^) predicting gene accumulation. Determines if a pangenome is “open” (γ > 0) or “closed” (γ < 0).	Evolutionary time scale HGT rates Niche versatility	Sampling depth: undersampling can mask openness. Population structure: oversampling clones mimics closure.
Pangenome fluidity	Metric estimating the gene content dissimilarity between random pairs of genomes. Robust to sample size variations.	Effective population size (*Ne*) Lifestyle (sympatric versus allopatric) Genome size	Representativeness: biased sampling alters this metric. Dereplication: redundancy (clones) artificially lowers fluidity.
Pangenome graphs	Network-based data model representing genomes as nodes and edges. Captures structural variation and synteny.	Genomic architecture Rearrangement rates Local gene order	Assembly quality: fragmented genomes break graph topology. Alignment thresholds: stringency affects node merging.

A species’ pangenome fluidity is strongly correlated with its lifestyle. The fundamental principle is that more variable lifestyles are associated with more fluid (i.e. more open) pangenomes (Kislyuk *et al.* 2011, Shapiro 2017, Tonkin-Hill *et al.* 2023) This pattern is observable across multiple ecological axes; For instance, free-living bacteria, which encounter a wide array of environmental conditions and microbial partners, consistently exhibit more fluid pangenomes than species strictly associated with hosts (Tonkin-Hill *et al.* 2023). This trend continues within host-associated bacteria, facultative symbionts have more fluid pangenomes than obligate ones, extracellular species more than intracellular specialists, and mutualists more than pathogens (Tonkin-Hill *et al.* 2023).

**Figure 5:**
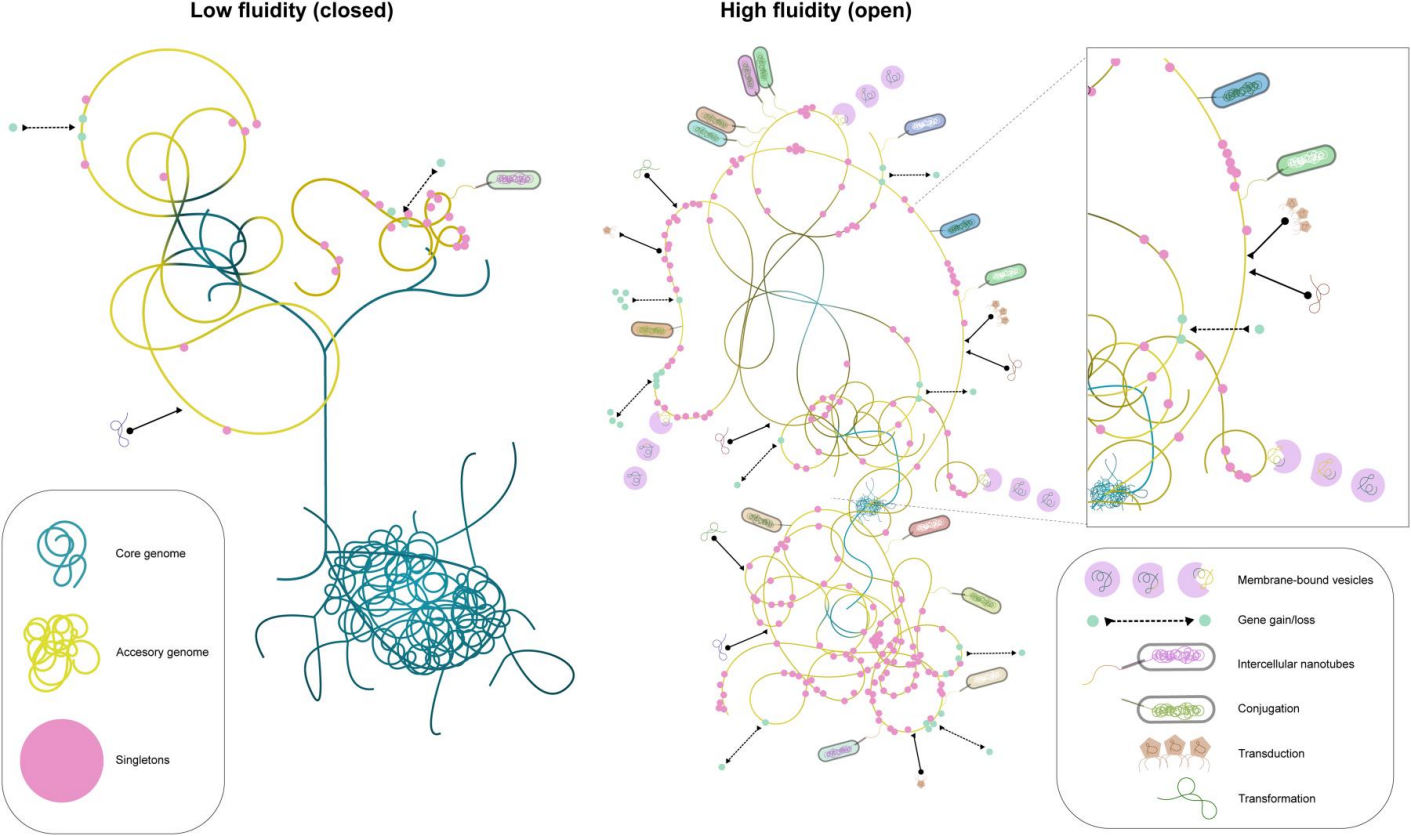
The pangenome fluidity spectrum driven by horizontal gene transfer (HGT). (A) Low fluidity (closed) pangenome characterized by a large, stable core genome and a limited accessory genome. (B) High fluidity (open) pangenome displaying a reduced core genome, an expansive accessory genome, and numerous singletons. The core genome is shown in blue, the accessory genome in yellow, and singletons in pink. The inset box (top right) provides a magnified view of the diverse HGT mechanisms that drive pangenome fluidity, including transformation, transduction, conjugation, membrane-bound vesicles, and intercellular nanotubes.

The taxa from our previous analysis (Figs 4 and 5) can be re-contextualized through this lens. The open pangenomes of the environmental generalists *Chromobacterium* and *Collimonas*, and the gut-dweller *Methanobrevibacter*, reflect high fluidity. This is driven by their exposure to diverse environments and frequent HGT, resulting in expansive accessory genomes. In contrast, the closed pangenomes of the endosymbionts *Candidatus Liberibacter asiaticus* and *Candidatus Atelocyanobacterium* exemplify low-fluidity pangenomes, a hallmark of genomic stability in isolated, specialized niches.

This relationship is driven by the balance between gene gain and loss (Tonkin-Hill *et al.* 2023). Prokaryotes in ecologically diverse, or sympatric, settings are exposed to a larger pool of mobile genetic elements (the “mobilome”), leading to frequent gene gain events (Rouli *et al.* 2015, Tettelin and Medini 2020). This constant influx of genetic material, which includes phages, plasmids, and transposons, contributes to a larger, more dynamic pangenome (Fig. 5). These organisms often co-evolve more extensive defense mechanisms, such as CRISPR-Cas systems, in response to this high rate of genetic exchange (Rouli *et al.* 2015). Conversely, species occupying restricted and stable niches, such as intracellular pathogens or endosymbionts living in allopatry, face reduced exposure to foreign DNA (Rouli *et al.* 2015, Tettelin and Medini 2020, Dewar *et al.* 2024). This results in smaller, more stable genomes and, consequently, a less fluid or “closed” pangenome (Fig. 5). The rate of gene gain and loss is therefore a critical dynamic that directly influences a species’ capacity to adapt. This adaptive potential, rooted in pangenome fluidity, is a key determinant of a prokaryote’s evolutionary trajectory (Tonkin-Hill *et al.* 2023).

While lifestyle is a principal driver, the influence of genome size and effective population size (*Ne*) presents a more complex picture. Both are generally found to be positively correlated with pangenome fluidity (Bobay and Ochman 2018, Maistrenko *et al.* 2020). A large *Ne* is fundamental for the adaptive evolution of pangenomes, as it can facilitate the acquisition and retention of advantageous genes (McInerney *et al.* 2017, Bobay and Ochman 2018). However, recent phylogenetic approaches challenge this view, suggesting that lifestyle is the most influential factor (Dewar *et al.* 2024). This study shown that while species with more variable lifestyles tend to have larger effective population sizes, *Ne* itself has little to no direct influence on pangenome fluidity (Dewar *et al.* 2024). Instead, its observed correlation may be an artifact of its co-variation with lifestyle. The same analysis confirms that while genome size has a direct influence, lifestyle remains the most significant determinant of pangenome fluidity (Dewar *et al.* 2024).

These ecological and population-level dynamics are mediated by the mechanisms of HGT, which are fundamental to creating the gene-content variation that defines pangenome fluidity. The flux of genes is mediated by processes including the uptake of naked DNA from the environment (transformation), transduction by bacteriophages, and direct cell-to-cell transfer via conjugative pili (Fig. 5) (Hall *et al.* 2017, McInerney *et al.* 2017, McInerney 2023). More recently discovered mechanisms, such as the exchange of membrane-bound vesicles and intercellular nanotubes, further contribute to this collective mobilome (Hall *et al.* 2017, McInerney *et al.* 2017, McInerney 2023). These HGT events, shaped by intra-genomic selective pressures, are fundamental to the origin, maintenance, and structuring of prokaryotic pangenomes, providing the genetic substrate for adaptation and diversification (McInerney 2023).

## 11 The pangenome in action: a world of innovative applications

The conceptual shift from a single reference genome to a pangenome has unlocked a vast array of practical applications, fundamentally reshaping our approach to microbiology and genetics. By capturing the complete genetic diversity of a species, this approach provides unprecedented resolution for understanding evolution, pathogenicity, and biotechnological potential.

In medicine and public health, pangenomics has become an indispensable tool for tracking the spread and evolution of pathogens. By analyzing the accessory genome, researchers can identify genes associated with antimicrobial resistance (AMR), virulence factors, and host immune evasion mechanisms. This has been critical in studying major pathogens such as *Neisseria meningitidis* (Yang *et al.* 2023), Streptococcus *pneumoniae* (Donati *et al.* 2010*)*, *Helicobacter pylori (*Gressmann *et al.* 2005*), Mycobacterium tuberculosis* (Kavvas *et al.* 2018*)*, *E. coli* (Tantoso *et al.* 2022), *Salmonella enterica* (Coluzzi *et al.* 2025), and *Staphylococcus aureus* (Macori *et al.* 2019).

Beyond surveillance, pangenomics is central to disease prevention through modern reverse vaccinology, a field that screens an organism’s entire gene repertoire to identify novel vaccine candidates. This strategy uncovers antigens missed by single-reference approaches and has been successfully applied to diverse pathogens including *Mycobacteroides abscessus* (Dar *et al.* 2021), *Leptospira interrogans* (Zeng *et al.* 2017), *Enterococcus faecium* (Alotaibi *et al.* 2022) *Stenotrophomonas maltophilia* (Shovon *et al.* 2024), and has even been extended to viruses like SARS-CoV-2 (Haseeb *et al.* 2022). Furthermore, the pangenome provides a rich blueprint for discovering new therapeutics. Essential genes within the core genome represent ideal targets for broad spectrum antibiotics, as they are indispensable for survival (Caicedo-Montoya *et al.* 2021), while the diverse accessory genome offers a vast reservoir of novel biosynthetic gene clusters that may produce molecules with antibiotic activity (Khan *et al.* 2023).

The pangenome framework is also transforming biotechnology and fundamental research. It enables the identification of novel enzymes and metabolic pathways in microorganisms of industrial interest from diverse environments, including species of *Delftia*, *Pedobacter*, *Pseudoalteromonas*, *Flavobacterium*, Lactobacillaceae, and various extremophiles (Bosi *et al.* 2017, Gaba *et al.* 2020, Yin *et al.* 2022, Covas *et al.* 2023, Kim *et al.* 2023, Ardalani *et al.* 2024, Mol and de Maayer 2024). It is also a powerful tool for studying microbial defense mechanisms, such as CRISPR-Cas and restriction-modification systems (Doron *et al.* 2018), and for identifying novel genomic islands that are often indicated by unique gene clusters (Yang and Gao 2022).

Methodologically, by overcoming the reference bias inherent in single-genome analyses, the pangenome approach enables the accurate quantification of reads from variable genes, thus capturing a more comprehensive snapshot of gene expression in transcriptomic studies (Chaves-Moreno *et al.* 2015, Tong *et al.* 2025). Moreover, pangenomics offers a robust framework for prokaryotic systematics, addressing the challenges of traditional prokaryotic classification methods that rely on a small number of marker genes. By using the stable, conserved core genome, high-resolution phylogenomic trees can be constructed, which has been instrumental in the formal reclassification of numerous prokaryotic taxa (Caputo *et al.* 2015, 2019).

Although pioneered in prokaryotes, pangenomics is now being successfully applied to eukaryotes. Human pangenome projects have revealed vast amounts of previously uncharacterized structural variation and incorporated hundreds of megabases of novel DNA sequences, creating a more complete and representative reference for human genetic diversity (Gao *et al.* 2023, Taylor *et al.* 2024). In agriculture, studies of various plants have identified numerous genes related to important agronomic traits, such as tomato flavor, while also uncovering the reduction in genetic diversity caused by domestication (Gao *et al.* 2019, Cochetel *et al.* 2023, Wang *et al.* 2023, Jayakodi *et al.* 2024, Kaur *et al.* 2024). Similarly, its application to animals is also a rapidly advancing field (Zhou *et al.* 2022, Rice *et al.* 2023, Hickey *et al.* 2024, Miao *et al.* 2024, 2025). However, applying pangenomics to eukaryotes presents unique difficulties due to their larger and more complex genomes, which necessitates the continuous design of more sophisticated analytical methodologies.

## 12 Pangenomics of the third domain (Archaea): an under-explored

**Figure 6:**
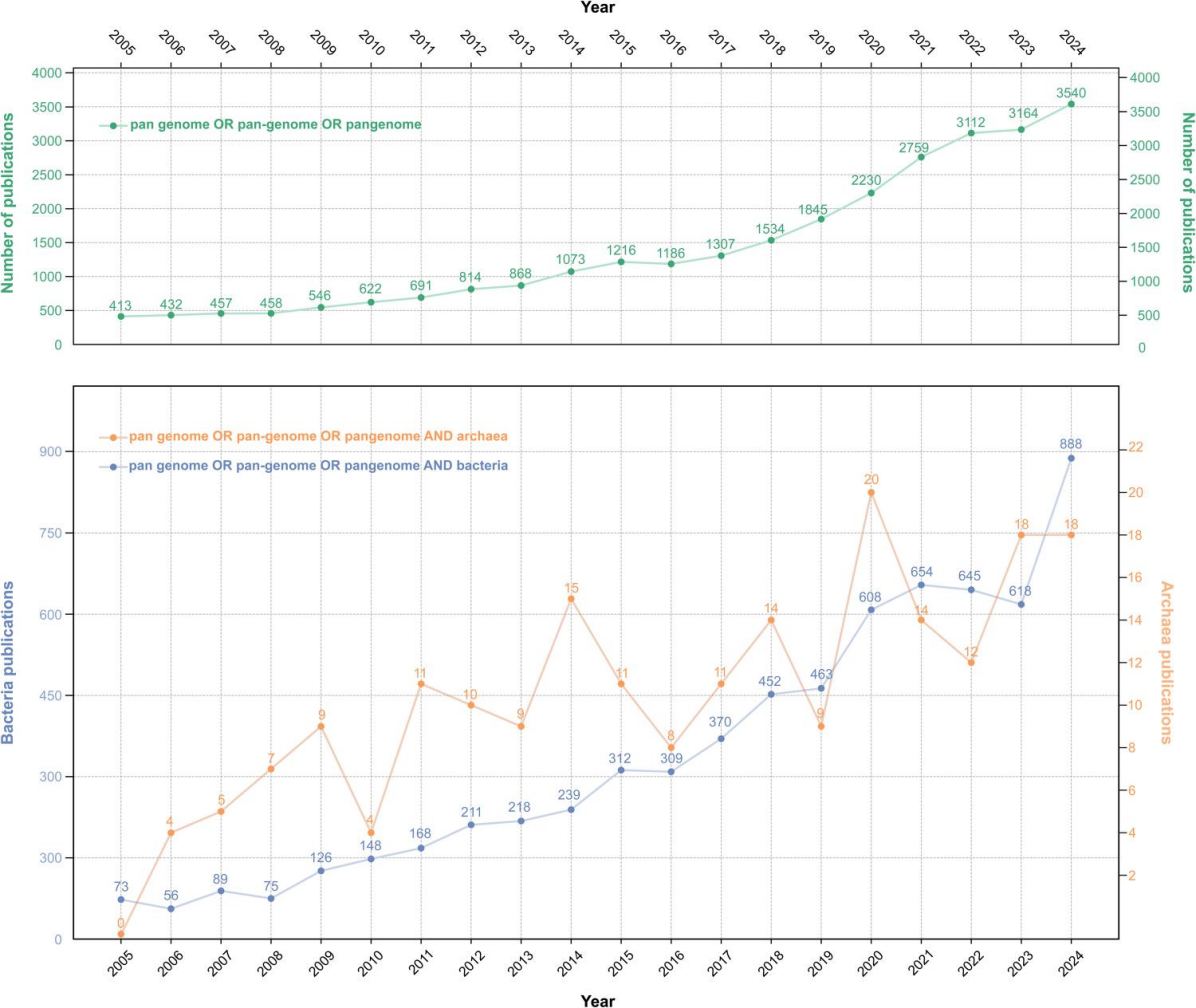
Publication trends for pangenome studies in the NCBI PubMed database (2005–2024). This two-panel graph illustrates the annual evolution in the number of scientific publications related to the search terms “pangenome,” “pan-genome,” or “pan genome,” as retrieved from the NCBI PubMed database. The top panel shows the results of a general search for these terms (grey line), reflecting the overall growth of the field. The bottom panel presents a detailed comparison using a dual *Y*-axis format to visualize the two prokaryotic domains with different numerical scales: the blue line (left *Y*-axis) quantifies studies on Bacteria pangenomes, while the orange line (right *Y*-axis) quantifies studies on Archaea pangenomes. All data points across the figure are labeled with their precise numerical values. The figure highlights both the exponential growth in pangenome research and the notable disparity in the volume of studies between the bacterial and archaeal domains documented in PubMed.

The exploration of archaeal pangenomes, while yielding significant insights, has historically lagged behind that of their bacterial counterparts. This disparity can be primarily attributed to two key factors: the relatively recent recognition of Archaea as a distinct domain of life and the considerable challenges associated with cultivating most archaeal lineages (Balch *et al.* 1977, Woese and Fox 1977, Springer *et al.* 1995, Rafiq *et al.* 2023). A large proportion of archaeal diversity remains uncultured, presenting significant hurdles such as long generation times or complex syntrophic dependencies, which has limited the availability of high-quality genomes (Baker *et al.* 2020, Padalko *et al.* 2024). This is reflected in the genomic data itself: with only ∼30 565 archaeal genomes available compared to over 2.68 million for Bacteria, the data for Bacteria is nearly 90× more abundant (Fig. 3). Furthermore, while the number of pangenome publications has grown exponentially for Bacteria, studies on Archaea have remained inconsistent and show no clear upward trend (Fig. 6). Consequently, our understanding of the full scope of archaeal genetic diversity is still evolving.

Despite these challenges, pangenomic studies in specific archaeal groups have revealed remarkable adaptive strategies (Table 2). In halophilic archaea, for instance, the genus *Halobacterium* exhibits an open pangenome rich with genes for ecological specialization, including those for sulfolipid biosynthesis (for membrane stability) and light-sensing mechanisms like bacteriorhodopsin (Medina-Chávez *et al.* 2025). This showcases genomic adaptation to specific, harsh conditions. Even more intriguing are findings in *Halorubrum ezzemoulense*, where analysis uncovered novel genomic structures termed “homeocassettes”—genomic islands with divergent gene content at conserved insertion sites, suggesting sophisticated genome management beyond simple gene gain or loss (Feng *et al.* 2024).

**Table 2:** Comparative overview of pangenome studies in diverse archaeal taxa.

Taxonomic group	Phylum	No. genomes	Nature	Core genes	Total genes	References
Class Halobacteria	Methanobacteriota	111	Open	300	49.613	Gaba *et al.* (2020)
Euryarchaeota	Methanobacteriota	430	Open	45	60.809	Gaba *et al.* (2020)
*Halorubrum ezzemoulense*	Methanobacteriota	47	N/A	2.621	8.401	Feng *et al.* (2024)
Genus *Haloarcula*	Methanobacteriota	32	Open	690	13.782	Verma *et al.* (2020)
Genus *Halomicrobium*	Methanobacteriota	5	Open	3.551	6.129	Verma *et al.* (2020)
Genus *Halarchaeum*	Methanobacteriota	6	N/A	N/A	>13.576	Wang *et al.* (2024)
*Halobacterium salinarum*	Methanobacteriota	35	Open	1.072	3.744	Medina-Chávez *et al.* (2025)
Haloferacaceae	Methanobacteriota	106	Open	N/A	N/A	Griffiths *et al.* (2024)
Cultured methanogens	Methanobacteriota	86	N/A	552’	10.131’	Prondzinsky *et al.* (2023)
*Methanobrevibacter smithii*	Methanobacteriota	23	N/A	987	2.847	Hansen *et al.* (2011)
*Methanopyrus kandleri*	Methanobacteriota	3	Closed	1.404	1.959	Yu *et al.* (2017)
*Methanococcus maripaludis*	Methanobacteriota	21	Open	1.417	3.552	Li *et al.* (2023)
Order Thermococcales	Methanobacteriota	30	Open	773	6.070	Zhong *et al.* (2021)
Euryarchaeota GII/III	Methanobacteriota	452	N/A	704	3.527	Deschamps *et al.* (2014)
*Sulfolobus islandicus*	Thermoproteota	7	N/A	2.169	2.931	Reno *et al.* (2009)
Genus *Metallosphaera*	Thermoproteota	19	Open	85	6.499	Wang *et al.* (2020)
Thaumarchaeota	Nitrososphaerota	545	N/A	706	2.098	Deschamps *et al.* (2014)
Genus *Nitrosopumilus*	Nitrososphaerota	N/A	Open	1.097	4.094	Kamanda Ngugi *et al.* (2015)
Nanoarchaea	Nanobdellota	11	Open	N/A	5.840	Hassani *et al.* (2024)
Asgard archaea	Promethearchaeota	936	Open	N/A	N/A	Köstlbacher *et al.* (2024)

Analyses of methanogenic archaea pangenome have revealed significant environmental influences on genome architecture and unique adaptive capabilities. Across a diverse group of 86 methanogen species, a surprisingly large conserved genomic core was identified, indicating that their shared physiology imposes strong evolutionary constraints (Prondzinsky *et al.* 2023). A key finding from this research is the correlation between increased growth temperature and both a reduction in overall genome size and an increase in the core genome’s proportion, suggesting temperature as a potent selective pressure shaping their genomes (Prondzinsky *et al.* 2023). Within the *Methanosarcina* genus, comparative studies have revealed considerable genomic plasticity; Methanosarcina *barkeri* shows significant disorder in its distal genome regions and possesses unique adaptive genes not found in close relatives, such as those for a complete formate dehydrogenase operon and gas vesicles, pointing to distinct evolutionary paths and niche specializations (Maeder *et al.* 2006). Furthermore, analysis of genomes from the *Methanomassiliicoccales* order, typically associated with animal digestive tracts, revealed adaptations for a free-living lifestyle, including genes for agmatine production (pH regulation), trehalose biosynthesis (osmotic/temperature regulation) and arsenic detoxification, thereby expanding our understanding of their ecological versatility (Cozannet *et al.* 2020).

In thermophilic archaea like *Sulfolobus*, pangenomics has clearly demonstrated that their accessory genomes are enriched with genes conferring metabolic versatility, particularly for nitrogen and sulfur metabolism (Dai *et al.* 2016). Beyond these lineage-specific insights, archaeal pangenomics has uncovered broader evolutionary phenomena that challenge traditional views. A striking example is the identification of genes for pseudomurein biosynthesis in some methanogens, which are of bacterial origin (Lupo *et al.* 2025). This discovery of inter-domain HGT for components of the cell wall blurs the lines of domain-specific traits and impacts our understanding of the Last Archaeal Common Ancestor’s cell architecture (Lupo *et al.* 2025). Another significant finding is the prevalence of protein fusion and fission events as a distinct mechanism of genomic and functional innovation in Archaea, with over 162 such protein families identified as archaeal-specific (Padalko *et al.* 2024).

These results underscore that archaeal pangenomes harbor unique evolutionary narratives and sophisticated adaptive strategies. Therefore, despite the current reliance on a large proportion of MAGs and the recognized scarcity of genomes from cultivated isolates, the continued exploration of archaeal pangenomes is of critical importance. Such research is crucial for significantly advancing our knowledge of archaeal genetic diversity, uncovering novel biological functions with considerable biotechnological potential, and providing deeper insights into the evolutionary innovations that define this unique domain of life.

## 13 Future insights

The future of pangenomics lies in moving beyond linear representations of gene presence and absence towards graph-based models that embrace the full complexity of genomic variation. While foundational, traditional pangenomes fail to capture critical information such as structural variants, single nucleotide polymorphisms, and gene order. The widespread adoption of pangenome graphs represents the next logical leap. These approaches can be broadly categorized into gene-based graphs, which model gene adjacencies and functional modules to correct annotation errors and classify gene persistence (e.g. PPanGGOLiN, Panaroo) (Gautreau *et al.* 2020, Tonkin-Hill *et al.* 2020), and sequence-based graphs, which integrate nucleotide-level variation into a single, comprehensive reference structure. This shift will not only revolutionize re-sequencing analysis by eliminating reference bias but will also empower more robust analyses of genomic architecture.

The exponential growth in genomic data necessitates a parallel revolution in analytical methods, a role that artificial intelligence (AI) is uniquely poised to fill. As pangenomes scale to millions of genomes, traditional statistical approaches become computationally intractable and often fail to capture complex, non-linear interactions. Machine learning will become central to extracting biological meaning from this complexity, particularly in two key areas: phenotype prediction and functional annotation.

Advanced models will power a new generation of Pangenome-Wide Association Studies (PWAS). Unlike traditional Genome-Wide Association Studies (GWAS) that focus on single nucleotide polymorphisms (SNPs), PWAS correlates the presence or absence of accessory genes (and structural variants) with specific phenotypes (Brynildsrud *et al.* 2016, Manuweera *et al.* 2019, Roder *et al.* 2024), offering a more direct link to the causal mechanisms of traits like antimicrobial resistance or host specificity. Furthermore, AI offers a path to illuminating the function of the “microbial dark matter” (Zha *et al.* 2022, Zhang *et al.* 2024). AI models are being developed to learn the “language” of protein sequences, enabling functional predictions at an unprecedented scale and accuracy (Lin *et al.* 2023, Torres *et al.* 2025), thereby turning pangenomic data into actionable biological knowledge.

Pangenome analyses, even those utilizing sophisticated graph-based models, have inherent limitations in predicting complex phenotypes. The static genomic repertoire does not account for gene regulation, post-transcriptional modifications, or metabolic interactions. Consequently, the presence of a gene (or structural variant) does not guarantee its expression. To fully bridge the genotype–phenotype gap, the field must integrate pangenomics with complementary functional approaches. Concepts such as the pan-transcriptome—which captures the complete range of gene expression profiles across a taxon (Manuweera *et al.* 2019, Bhatti *et al.* 2020)—and the pan-metabolome—which encompasses the aggregate metabolic output and chemical diversity of a taxon (Rees *et al.* 2018, Bao and Liu 2020)—are becoming essential to connect gene content with actual cellular function and environmental adaptation.

The next frontier for pangenomics is to transcend the single-species level and embrace a community-wide perspective. The concept of the metapangenome—defined as the outcome of pangenomic analysis applied to metagenomic data to capture the population-level diversity of specific lineages within complex communities—will become central (Delmont and Eren 2018). This approach integrates pangenomics with metagenomics, allowing us to study the dynamics of gene flow, horizontal gene transfer, and adaptation not just within a species, but between species in a complex ecosystem.

Moreover, coupling these metapangenomic frameworks with metatranscriptomics and metametabolomics will be crucial to unravel not just which genes are present, but which are actively expressed and determining the chemical landscape of the ecosystem. Understanding these multi-layered dynamics will be key to deciphering how entire microbial communities respond to selective pressures, such as antibiotics or pollutants. Ultimately, the conceptual and computational frameworks pioneered in prokaryotes are now paving the way for tackling the even greater complexities of eukaryotic pangenomes, from humans to crops, marking the next major frontier for the field.

## 14 Conclusions

The pangenomic era has fundamentally shifted our perspective on microbial genomics, moving us beyond the static, single-genome paradigm to embrace the full spectrum of a taxon’s genetic diversity. This review has charted the evolution of pangenomics from its conceptual origins to its modern applications, highlighting the critical interplay between biological concepts like pangenome fluidity and the statistical frameworks used to model them.

Critically, a pangenome is not a fixed biological entity but rather a statistical model whose outputs are dependent on the underlying dataset. The quality, quantity, diversity, and taxonomic resolution of the input genomes are not mere technical details; they are the principal determinants shaping our interpretation of a pangenome’s size, its open or closed nature, and its functional potential. Understanding this principle is essential for moving the field towards more robust, reproducible, and meaningful biological insights.

Furthermore, the exponential expansion of modern datasets necessitates parallel advancements in data accessibility. To translate pangenomic complexity into actionable knowledge, the field must prioritize the development of scalable indexing methods for rapid sequence querying and interactive visualization tools that allow researchers to intuitively explore these vast gene pools.

As we advance further in this field, particularly into under-explored organisms like Archaea, a critical awareness of these methodological foundations is paramount. The future of pangenomics will be defined not just by the discovery of new genes, but by our ability to build sophisticated, ecologically informed, and statistically robust models that can accurately translate the vastness of genomic data into a true understanding of microbial life.

## Supplementary Material

vbag069_Supplementary_Data

## Data Availability

All data generated or analyzed during this study are included in this published article and its supplementary information files.

## References

[vbag069-B1] Albertsen M , Hugenholtz P, Skarshewski A et al Genome sequences of rare, uncultured bacteria obtained by differential coverage binning of multiple metagenomes. Nat Biotechnol 2013;31:533–8. 10.1038/nbt.2579 23707974

[vbag069-B2] Alotaibi G , Khan K, Al Mouslem AK et al Pan genome based reverse vaccinology approach to explore *Enterococcus faecium* (VRE) strains for identification of novel multi-epitopes vaccine candidate. Immunobiology 2022;227:152221. 10.1016/j.imbio.2022.152221 35483110

[vbag069-B3] Amann RI , Ludwig W, Schleifer KH. Phylogenetic identification and in situ detection of individual microbial cells without cultivation. Microbiol Rev 1995;59:143–69. 10.1128/mr.59.1.143-169.1995 7535888 PMC239358

[vbag069-B4] Anantharaman K , Brown CT, Hug LA et al Thousands of microbial genomes shed light on interconnected biogeochemical processes in an aquifer system. Nat Commun 2016;7:13219. 10.1038/ncomms13219 27774985 PMC5079060

[vbag069-B5] Ardalani O , Phaneuf PV, Mohite OS et al Pangenome reconstruction of *lactobacillaceae* metabolism predicts species-specific metabolic traits. MSystems 2024;9:e0015624. 10.1128/msystems.00156-24 38920366 PMC11265412

[vbag069-B6] Baker BJ , De Anda V, Seitz KW et al Diversity, ecology and evolution of archaea. Nat Microbiol 2020;5:887–900. 10.1038/s41564-020-0715-z 32367054

[vbag069-B7] Balch WE , Magrum LJ, Fox GE et al An ancient divergence among the bacteria. J Mol Evol 1977;9:305–11. 10.1007/BF01796092 408502

[vbag069-B8] Bao L , Liu X. Pan-metabolomics and its applications. In: Barh D, Soares SC, Tiwari S, Azevedo VADC (eds.), Pan-Genomics: Applications, Challenges, and Future Prospects. San Diego, CA: Elsevier/Academic Press, 2020, 371–95. 10.1016/B978-0-12-817076-2.00020-2

[vbag069-B9] Bhatti A , Shah FS, Azhar J et al Pan-transcriptomics and its applications. In: Barh D, Soares SC, Tiwari S, Azevedo VADC (eds.), Pan-Genomics: Applications, Challenges, and Future Prospects. San Diego, CA: Elsevier/Academic Press, 2020, 343–56. 10.1016/B978-0-12-817076-2.00018-4

[vbag069-B10] Bobay L-M , Ochman H. Factors driving effective population size and pan-genome evolution in bacteria. BMC Evol Biol 2018;18:153. 10.1186/s12862-018-1272-4 30314447 PMC6186134

[vbag069-B11] Bornstein K , Gryan G, Chang ES et al The NIH comparative genomics resource: addressing the promises and challenges of comparative genomics on human health. BMC Genomics 2023;24:575. 10.1186/s12864-023-09643-4 37759191 PMC10523801

[vbag069-B12] Bosi E , Fondi M, Orlandini V et al The pangenome of (antarctic) pseudoalteromonas bacteria: evolutionary and functional insights. BMC Genomics 2017;18:93. 10.1186/s12864-016-3382-y 28095778 PMC5240218

[vbag069-B13] Boulton W , Salamov A, Grigoriev IV et al Metagenome-assembled-genomes recovered from the arctic drift expedition MOSAiC. Sci Data 2025;12:204. 10.1038/s41597-025-04525-8 39904998 PMC11794607

[vbag069-B14] Brown CT , Hug LA, Thomas BC et al Unusual biology across a group comprising more than 15% of domain bacteria. Nature 2015;523:208–11. 10.1038/nature14486 26083755

[vbag069-B15] Brynildsrud O , Bohlin J, Scheffer L et al Rapid scoring of genes in microbial pan-genome-wide association studies with scoary. Genome Biol 2016;17:238. 10.1186/s13059-016-1108-8 27887642 PMC5124306

[vbag069-B16] Caicedo-Montoya C , Manzo-Ruiz M, Ríos-Estepa R. Pan-genome of the genus streptomyces and prioritization of biosynthetic gene clusters with potential to produce antibiotic compounds. Front Microbiol 2021;12:677558. 10.3389/fmicb.2021.677558 34659136 PMC8510958

[vbag069-B17] Caputo A , Fournier P-E, Raoult D. Genome and pan-genome analysis to classify emerging bacteria. Biol Direct 2019;14:5. 10.1186/s13062-019-0234-0 30808378 PMC6390601

[vbag069-B18] Caputo A , Merhej V, Georgiades K et al Pan-genomic analysis to redefine species and subspecies based on quantum discontinuous variation: the Klebsiella paradigm. Biol Direct 2015;10:55. 10.1186/s13062-015-0085-2 26420254 PMC4588269

[vbag069-B19] Carlos Guimaraes L , Benevides de Jesus L, Vinicius Canario Viana M et al Inside the pan-genome - methods and software overview. Curr Genomics 2015;16:245–52. 10.2174/1389202916666150423002311 27006628 PMC4765519

[vbag069-B20] Carpi FM , Coman MM, Silvi S et al Comprehensive pan‐genome analysis of *Lactiplantibacillus plantarum* complete genomes. J Appl Microbiol 2022;132:592–604. 10.1111/jam.15199 34216519 PMC9290807

[vbag069-B21] Caugant DA , Levin BR, Selander RK. Genetic diversity and temporal variation in the *E. coli* population of a human host. Genetics 1981;98:467–90. 10.1093/genetics/98.3.467 7037535 PMC1214454

[vbag069-B22] Chauhan SM , Ardalani O, Hyun JC et al Decomposition of the pangenome matrix reveals a structure in gene distribution in the *Escherichia coli* species. MSphere 2025;10:e0053224. 10.1128/msphere.00532-24 39745367 PMC11774025

[vbag069-B23] Chaves-Moreno D , Wos-Oxley ML, Jáuregui R et al Application of a novel “pan-genome”-based strategy for assigning RNAseq transcript reads to *Staphylococcus aureus* strains. PLoS One 2015;10:e0145861. 10.1371/journal.pone.0145861 26717500 PMC4696825

[vbag069-B24] Chávez-Luzanía RA , Montoya-Martínez AC, Parra-Cota FI et al Pangenomes-identified singletons for designing specific primers to identify bacterial strains in a plant growth-promoting consortium. Mol Biol Rep 2022;49:10489–98. 10.1007/s11033-022-07927-8 36125674

[vbag069-B25] Chen L-X , Anantharaman K, Shaiber A et al Accurate and complete genomes from metagenomes. Genome Res 2020;30:315–33. 10.1101/gr.258640.119 32188701 PMC7111523

[vbag069-B26] Chen Y-H , Chiang P-W, Rogozin DY et al Salvaging high-quality genomes of microbial species from a meromictic lake using a hybrid sequencing approach. Commun Biol 2021;4:996. 10.1038/s42003-021-02510-6 34426638 PMC8382752

[vbag069-B27] Chibani CM , Mahnert A, Borrel G et al A catalogue of 1,167 genomes from the human gut archaeome. Nat Microbiol 2022;7:48–61. 10.1038/s41564-021-01020-9 34969981 PMC8727293

[vbag069-B28] Chklovski A , Parks DH, Woodcroft BJ et al CheckM2: a rapid, scalable and accurate tool for assessing microbial genome quality using machine learning. Nat Methods 2023;20:1203–12. 10.1038/s41592-023-01940-w 37500759

[vbag069-B29] Cochetel N , Minio A, Guarracino A et al A super-pangenome of the North American wild grape species. Genome Biol 2023;24:290. 10.1186/s13059-023-03133-2 38111050 PMC10729490

[vbag069-B30] Coluzzi C , Piscon B, Dérozier S et al Comparative genomics of *Salmonella enterica* serovars paratyphi A, typhi and typhimurium reveals distinct profiles of their pangenome, mobile genetic elements, antimicrobial resistance and defense systems repertoire. Virulence 2025;16:2504658. 10.1080/21505594.2025.2504658 40394957 PMC12101602

[vbag069-B31] Colwell RR. Microbial diversity: the importance of exploration and conservation. J Ind Microbiol Biotechnol 1997;18:302–7. 10.1038/sj.jim.2900390 9218360

[vbag069-B32] Cooley NP , Wright ES. Many purported pseudogenes in bacterial genomes are bona fide genes. BMC Genomics 2024;25:365. 10.1186/s12864-024-10137-0 38622536 PMC11017572

[vbag069-B33] Cordero OX , Polz MF. Explaining microbial genomic diversity in light of evolutionary ecology. Nat Rev Microbiol 2014;12:263–73. 10.1038/nrmicro3218 24590245

[vbag069-B34] Covas C , Figueiredo G, Gomes M et al The pangenome of gram-negative environmental bacteria hides a promising biotechnological potential. Microorganisms 2023;11:2445. 10.3390/microorganisms11102445 37894103 PMC10609062

[vbag069-B35] Cox E , Tsuchiya MTN, Ciufo S et al NCBI taxonomy: enhanced access via NCBI datasets. Nucleic Acids Res 2025;53:D1711–5. 10.1093/nar/gkae967 39470745 PMC11701650

[vbag069-B36] Cozannet M , Borrel G, Roussel E et al New insights into the ecology and physiology of methanomassiliicoccales from terrestrial and aquatic environments. Microorganisms 2020;9:30. 10.3390/microorganisms9010030 33374130 PMC7824343

[vbag069-B37] Cummins EA , Hall RJ, McInerney JO et al Prokaryote pangenomes are dynamic entities. Curr Opin Microbiol 2022;66:73–8. 10.1016/j.mib.2022.01.005 35104691

[vbag069-B38] Dai X , Wang H, Zhang Z et al Genome sequencing of sulfolobus sp. A20 from Costa Rica and comparative analyses of the putative pathways of carbon, nitrogen, and sulfur metabolism in various sulfolobus strains. Front Microbiol 2016;7:1902. 10.3389/fmicb.2016.01902 27965637 PMC5127849

[vbag069-B39] Danneels B , Pinto-Carbó M, Carlier A. Patterns of nucleotide deletion and insertion inferred from bacterial pseudogenes. Genome Biol Evol 2018;10:1792–802. 10.1093/gbe/evy140 29982456 PMC6054270

[vbag069-B40] Dar HA , Ismail S, Waheed Y et al Designing a multi-epitope vaccine against *Mycobacteroides abscessus* by pangenome-reverse vaccinology. Sci Rep 2021;11:11197. 10.1038/s41598-021-90868-2 34045649 PMC8159972

[vbag069-B41] Darolt JC , Bento F. D M, Merlin BL et al The genome of “candidatus liberibacter asiaticus” is highly transcribed when infecting the gut of *Diaphorina citri*. Front Microbiol 2021;12:687725. 10.3389/fmicb.2021.687725 34322103 PMC8312247

[vbag069-B42] D’Auria G , Jiménez-Hernández N, Peris-Bondia F et al Legionella pneumophila pangenome reveals strain-specific virulence factors. BMC Genomics 2010;11:181. 10.1186/1471-2164-11-181 20236513 PMC2859405

[vbag069-B43] De Crécy-Lagard V , Hanson A. Comparative genomics. In: Brenner’s Encyclopedia of Genetics. San Diego, CA: Elsevier/Academic Press, 2013, 102–5. 10.1016/B978-0-12-374984-0.00299-0

[vbag069-B44] Delmont TO , Eren AM. Linking pangenomes and metagenomes: the *prochlorococcus* metapangenome. PeerJ 2018;6:e4320. 10.7717/peerj.4320 29423345 PMC5804319

[vbag069-B280] Deschamps P , Zivanovic Y, Moreira D et al Pangenome evidence for extensive interdomain horizontal transfer affecting lineage core and shell genes in uncultured planktonic thaumarchaeota and euryarchaeota. Genome Biol Evol 2014;6:1549–63. 10.1093/gbe/evu127 24923324 PMC4122925

[vbag069-B45] Dewar AE , Hao C, Belcher LJ et al Bacterial lifestyle shapes pangenomes. Proc Natl Acad Sci USA 2024;121:e2320170121. 10.1073/pnas.2320170121 PMC11126918 38743630

[vbag069-B46] Dimitrova PD , Damyanova T, Paunova-Krasteva T. *Chromobacterium violaceum*: a model for evaluating the anti-quorum sensing activities of plant substances. Sci Pharm 2023;91:33. 10.3390/scipharm91030033

[vbag069-B47] Domingo-Sananes MR , McInerney JO. Mechanisms that shape microbial pangenomes. Trends Microbiol 2021;29:493–503. 10.1016/j.tim.2020.12.004 33423895

[vbag069-B48] Donati C , Hiller NL, Tettelin H et al Structure and dynamics of the pan-genome of *Streptococcus pneumoniae* and closely related species. Genome Biol 2010;11:R107. 10.1186/gb-2010-11-10-r107 21034474 PMC3218663

[vbag069-B49] Doron S , Melamed S, Ofir G et al Systematic discovery of antiphage defense systems in the microbial pangenome. Science 2018;359:eaar4120. 10.1126/science.aar4120 29371424 PMC6387622

[vbag069-B50] Douglas GM , Shapiro BJ. Pseudogenes act as a neutral reference for detecting selection in prokaryotic pangenomes. Nat Ecol Evol 2024;8:304–14. 10.1038/s41559-023-02268-6 38177690

[vbag069-B51] Eisenhofer R , Odriozola I, Alberdi A. Impact of microbial genome completeness on metagenomic functional inference. ISME Commun 2023;3:12. 10.1038/s43705-023-00221-z 36797336 PMC9935889

[vbag069-B52] Eizenga JM , Novak AM, Sibbesen JA et al Pangenome graphs. Annu Rev Genomics Hum Genet 2020;21:139–62. 10.1146/annurev-genom-120219-080406 32453966 PMC8006571

[vbag069-B53] Feng Y , Arsenault D, Louyakis AS et al Using the pan-genomic framework for the discovery of genomic islands in the haloarchaeon *Halorubrum ezzemoulense*. MBio 2024;15:e0040824. 10.1128/mbio.00408-24 38619241 PMC11078007

[vbag069-B54] Gaba S , Kumari A, Medema M et al Pan-genome analysis and ancestral state reconstruction of class halobacteria: probability of a new super-order. Sci Rep 2020;10:21205. 10.1038/s41598-020-77723-6 33273480 PMC7713125

[vbag069-B55] Gao L , Gonda I, Sun H et al The tomato pan-genome uncovers new genes and a rare allele regulating fruit flavor. Nat Genet 2019;51:1044–51. 10.1038/s41588-019-0410-2 31086351

[vbag069-B56] Gao Y , Yang X, Chen H et al; Chinese Pangenome Consortium (CPC). A pangenome reference of 36 Chinese populations. Nature 2023;619:112–21. 10.1038/s41586-023-06173-7 37316654 PMC10322713

[vbag069-B57] Gaston KJ. Global patterns in biodiversity. Nature 2000;405:220–7. 10.1038/35012228 10821282

[vbag069-B58] Gautreau G , Bazin A, Gachet M et al PPanGGOLiN: depicting microbial diversity via a partitioned pangenome graph. PLoS Comput Biol 2020;16:e1007732. 10.1371/journal.pcbi.1007732 32191703 PMC7108747

[vbag069-B59] Glaser P , Rusniok C, Buchrieser C et al Genome sequence of *Streptococcus agalactiae*, a pathogen causing invasive neonatal disease. Mol Microbiol 2002;45:1499–513. 10.1046/j.1365-2958.2002.03126.x 12354221

[vbag069-B60] Goussarov G , Claesen J, Mysara M et al Accurate prediction of metagenome-assembled genome completeness by MAGISTA, a random Forest model built on alignment-free intra-bin statistics. Environ Microbiome 2022;17:9. 10.1186/s40793-022-00403-7 35248155 PMC8898458

[vbag069-B61] Gressmann H , Linz B, Ghai R et al Gain and loss of multiple genes during the evolution of *Helicobacter pylori*. PLoS Genet 2005;1:e43. 10.1371/journal.pgen.0010043 16217547 PMC1245399

[vbag069-B240] Griffiths DB , Tiwari RP, Murphy DV et al Comparative genomics of the highly halophilic Haloferacaceae. Sci Rep 2024;14:27025. 10.1038/s41598-024-78438-8 39506039 PMC11541754

[vbag069-B62] Hall JPJ , Brockhurst MA, Harrison E. Sampling the mobile gene Pool: innovation via horizontal gene transfer in bacteria. Philos Trans R Soc Lond B Biol Sci 2017;372:20160424. 10.1098/rstb.2016.0424 29061896 PMC5665811

[vbag069-B250] Hansen EE , Lozupone CA, Rey FE et al Pan-genome of the dominant human gut-associated archaeon, Methanobrevibacter smithii, studied in twins. Proc Natl Acad Sci 2011;108(supplement_1):4599–606. 10.1073/pnas.1000071108 21317366 PMC3063581

[vbag069-B63] Haseeb M , Amir A, Ikram A. Pangenome analysis of SARS-Cov2 strains to identify potential vaccine targets by reverse vaccinology. J Bioinform Syst Biol 2022;5:144–57. 10.26502/jbsb.5107042

[vbag069-B310] Hassani Y , Aboudharam G, Drancourt M. et al The discovery of Candidatus Nanopusillus phoceensis sheds light on the diversity of the microbiota nanoarchaea. IScience 2024;27:109488. 10.1016/j.isci.2024.109488 38595798 PMC11001627

[vbag069-B64] Hickey G , Monlong J, Ebler J et al; Human Pangenome Reference Consortium. Pangenome graph construction from genome alignments with minigraph-cactus. Nat Biotechnol 2024;42:663–73. 10.1038/s41587-023-01793-w 37165083 PMC10638906

[vbag069-B65] Hugerth LW , Larsson J, Alneberg J et al Metagenome-assembled genomes uncover a global brackish microbiome. Genome Biol 2015;16:279. 10.1186/s13059-015-0834-7 26667648 PMC4699468

[vbag069-B66] Jain C , Rodriguez-R LM, Phillippy AM et al High throughput ANI analysis of 90K prokaryotic genomes reveals clear species boundaries. Nat Commun 2018;9:5114. 10.1038/s41467-018-07641-9 30504855 PMC6269478

[vbag069-B67] Jaiswal AK , Tiwari S, Jamal SB et al The pan-genome of *Treponema pallidum* reveals differences in genome plasticity between subspecies related to venereal and non-venereal syphilis. BMC Genomics 2020;21:33. 10.1186/s12864-019-6430-6 31924165 PMC6953169

[vbag069-B68] Jayakodi M , Lu Q, Pidon H et al Structural variation in the pangenome of wild and domesticated barley. Nature 2024;636:654–62. 10.1038/s41586-024-08187-1 39537924 PMC11655362

[vbag069-B300] Kamanda Ngugi D , Blom J, Alam I. et al Comparative genomics reveals adaptations of a halotolerant thaumarchaeon in the interfaces of brine pools in the Red Sea. ISME J 2015;9:396–411. 10.1038/ismej.2014.137 25105904 PMC4303633

[vbag069-B69] Karampatakis T , Tsergouli K, Behzadi P. Pan-genome plasticity and virulence factors: a natural treasure trove for *Acinetobacter baumannii*. Antibiotics (Basel) 2024;13:257. 10.3390/antibiotics13030257 38534692 PMC10967457

[vbag069-B70] Kaur H , Shannon LM, Samac DA. A stepwise guide for pangenome development in crop plants: an alfalfa (*Medicago sativa*) case study. BMC Genomics 2024;25:1022. 10.1186/s12864-024-10931-w 39482604 PMC11526573

[vbag069-B71] Kavvas ES , Catoiu E, Mih N et al Machine learning and structural analysis of *Mycobacterium tuberculosis* pan-genome identifies genetic signatures of antibiotic resistance. Nat Commun 2018;9:4306. 10.1038/s41467-018-06634-y 30333483 PMC6193043

[vbag069-B72] Kawashima T. Comparative and evolutionary genomics. In: Encyclopedia of Bioinformatics and Computational Biology. Oxford: Elsevier, 2019, 257–67. 10.1016/B978-0-12-809633-8.20236-7

[vbag069-B73] Khan K , Jalal K, Uddin R. Pangenome profiling of novel drug target against vancomycin-resistant *Enterococcus faecium*. J Biomol Struct Dyn 2023;41:15647–60. 10.1080/07391102.2023.2191134 36935100

[vbag069-B74] Kim M , Cha I-T, Lee K-E et al Pangenome analysis provides insights into the genetic diversity, metabolic versatility, and evolution of the genus *flavobacterium*. Microbiol Spectr 2023;11:e0100323. 10.1128/spectrum.01003-23 37594286 PMC10655711

[vbag069-B75] Kislyuk AO , Haegeman B, Bergman NH et al Genomic fluidity: an integrative view of gene diversity within microbial populations. BMC Genomics 2011;12:32. 10.1186/1471-2164-12-32 21232151 PMC3030549

[vbag069-B76] Koonin EV , Wolf YI. Genomics of bacteria and archaea: the emerging dynamic view of the prokaryotic world. Nucleic Acids Res 2008;36:6688–719. 10.1093/nar/gkn668 18948295 PMC2588523

[vbag069-B951] Köstlbacher S , van Hooff JJE, Panagiotou K et al Structure-based inference of eukaryotic complexity in Asgard archaea. BioRxiv 2024. 10.1101/2024.07.03.601958

[vbag069-B77] Kuo C-H , Moran NA, Ochman H. The consequences of genetic drift for bacterial genome complexity. Genome Res 2009;19:1450–4. 10.1101/gr.091785.109 19502381 PMC2720180

[vbag069-B78] Kuo C-H , Ochman H. The extinction dynamics of bacterial pseudogenes. PLoS Genet 2010;6:e1001050. 10.1371/journal.pgen.1001050 20700439 PMC2916853

[vbag069-B79] Land M , Hauser L, Jun S-R et al Insights from 20 years of bacterial genome sequencing. Funct Integr Genomics 2015;15:141–61. 10.1007/s10142-015-0433-4 25722247 PMC4361730

[vbag069-B80] Lebeurre J , Dahyot S, Diene S et al Comparative genome analysis of *Staphylococcus lugdunensis* shows clonal complex-dependent diversity of the putative virulence factor, ess/type VII locus. Front Microbiol 2019;10:2479. 10.3389/fmicb.2019.02479 31736914 PMC6834553

[vbag069-B81] Lee I , Ouk Kim Y, Park S-C et al OrthoANI: an improved algorithm and software for calculating average nucleotide identity. Int J Syst Evol Microbiol 2016;66:1100–3. 10.1099/ijsem.0.000760 26585518

[vbag069-B82] Levin BR , Stewart FM. The population biology of bacterial plasmids: *a priori* conditions for the existence of mobilizable nonconjugative factors. Genetics 1980;94:425–43. 10.1093/genetics/94.2.425 6248416 PMC1214150

[vbag069-B83] Li S , Lian W-H, Han J-R et al Capturing the microbial dark matter in desert soils using culturomics-based metagenomics and high-resolution analysis. NPJ Biofilms Microbiomes 2023;9:67. 10.1038/s41522-023-00439-8 37736746 PMC10516943

[vbag069-B84] Li T , Yin Y. Critical assessment of pan-genomic analysis of metagenome-assembled genomes. Brief Bioinform 2022;23:bbac413. 10.1093/bib/bbac413 36124775 PMC9677465

[vbag069-B85] Lin Z , Akin H, Rao R et al Evolutionary-scale prediction of atomic-level protein structure with a language model. Science 2023;379:1123–30. 10.1126/science.ade2574 36927031

[vbag069-B86] Lupo V , Roomans C, Royen E et al Identification and characterization of archaeal pseudomurein biosynthesis genes through pangenomics. MSystems 2025;10:e0140124. 10.1128/msystems.01401-24 39936904 PMC11915815

[vbag069-B87] Macori G , Bellio A, Bianchi DM et al Genome-wide profiling of enterotoxigenic Staphylococcus aureus strains used for the production of naturally contaminated cheeses. Genes (Basel) 2019;11:33. 10.3390/genes11010033 31892220 PMC7016664

[vbag069-B88] Maeder DL , Anderson I, Brettin TS et al The *Methanosarcina barkeri* genome: comparative analysis with *Methanosarcina acetivorans* and *Methanosarcina mazei* reveals extensive rearrangement within methanosarcinal genomes. J Bacteriol 2006;188:7922–31. 10.1128/JB.00810-06 16980466 PMC1636319

[vbag069-B89] Maiden MCJ , Bygraves JA, Feil E et al Multilocus sequence typing: a portable approach to the identification of clones within populations of pathogenic microorganisms. Proc Natl Acad Sci USA 1998;95:3140–5. 10.1073/pnas.95.6.3140 9501229 PMC19708

[vbag069-B90] Maistrenko OM , Mende DR, Luetge M et al Disentangling the impact of environmental and phylogenetic constraints on prokaryotic within-species diversity. ISME J 2020;14:1247–59. 10.1038/s41396-020-0600-z 32047279 PMC7174425

[vbag069-B91] Manuweera B , Mudge J, Kahanda I et al Pangenome-wide association studies with frequented regions. In: *Proceedings of the 10th ACM International Conference on Bioinformatics, Computational Biology and Health Informatics*, *Niagara Falls, NY, USA*. New York, NY: Association for Computing Machinery, 2019, 627–32. 10.1145/3307339.3343478

[vbag069-B92] Matthews CA , Watson-Haigh NS, Burton RA et al A gentle introduction to pangenomics. Brief Bioinform 2024;25:bbae588. 10.1093/bib/bbae588 39552065 PMC11570541

[vbag069-B93] McInerney JO. Prokaryotic pangenomes act as evolving ecosystems. Mol Biol Evol 2023;40:msac232. 10.1093/molbev/msac232 36288801 PMC9851318

[vbag069-B94] McInerney JO , McNally A, O’Connell MJ. Why prokaryotes have pangenomes. Nat Microbiol 2017;2:17040. 10.1038/nmicrobiol.2017.40 28350002

[vbag069-B95] Médigue C , Rouxel T, Vigier P et al Evidence for horizontal gene transfer in *Escherichia coli* speciation. J Mol Biol 1991;222:851–6. 10.1016/0022-2836(91)90575-Q 1762151

[vbag069-B96] Medina-Chávez NO , Rodriguez-Cruz UE, Souza V et al Salty secrets of Halobacterium salinarum AD88: a new archaeal ecotype isolated from *Cuatro cienegas* basin. BMC Genomics 2025;26:399. 10.1186/s12864-025-11550-9 40275130 PMC12023398

[vbag069-B97] Meziti A , Rodriguez-R LM, Hatt JK et al The reliability of metagenome-assembled genomes (MAGs) in representing natural populations: insights from comparing MAGs against isolate genomes derived from the same fecal sample. Appl Environ Microbiol 2021;87:e02593. 10.1128/AEM.02593-20 33452027 PMC8105024

[vbag069-B98] Miao J , Wang Q, Zhang Z et al Pangenome graph mitigates heterozygosity overestimation from mapping bias: a case study in Chinese indigenous pigs. BMC Biol 2025;23:89. 10.1186/s12915-025-02194-y 40140905 PMC11948684

[vbag069-B99] Miao J , Wei X, Cao C et al Pig pangenome graph reveals functional features of non-reference sequences. J Anim Sci Biotechnol 2024;15:32. 10.1186/s40104-023-00984-4 38389084 PMC10882747

[vbag069-B100] Milkman R. Electrophoretic variation in *Escherichia coli* from natural sources. Science 1973;182:1024–6. 10.1126/science.182.4116.1024 4584002

[vbag069-B101] Mol M , de Maayer P. Elucidating the biotechnological potential of the genera parageobacillus and saccharococcus through comparative genomic and pan-genome analysis. BMC Genomics 2024;25:723. 10.1186/s12864-024-10635-1 39054411 PMC11270796

[vbag069-B102] Mueller FB. AI (artificial intelligence) and hypertension research. Curr Hypertens Rep 2020;22:70. 10.1007/s11906-020-01068-8 32852654 PMC7450041

[vbag069-B103] Nature Ecology and Evolution. A panoply of pangenomes. Nat Ecol Evolution 2024;8:833–833. 10.1038/s41559-024-02421-9 38741009

[vbag069-B104] Olm MR , Brown CT, Brooks B et al dRep: a tool for fast and accurate genomic comparisons that enables improved genome recovery from metagenomes through de-replication. ISME J 2017;11:2864–8. 10.1038/ismej.2017.126 28742071 PMC5702732

[vbag069-B105] Ondov BD , Treangen TJ, Melsted P et al Mash: fast genome and metagenome distance estimation using MinHash. Genome Biol 2016;17:132. 10.1186/s13059-016-0997-x 27323842 PMC4915045

[vbag069-B106] Orakov A , Fullam A, Coelho LP et al GUNC: detection of chimerism and contamination in prokaryotic genomes. Genome Biol 2021;22:178. 10.1186/s13059-021-02393-0 34120611 PMC8201837

[vbag069-B107] Ørskov I , Ørskov F. Special O: k: h serotypes among enterotoxigenic *E. coli* strains from diarrhea in adults and children. Med Microbiol Immunol 1977;163:99–110. 10.1007/BF02121825 331065

[vbag069-B108] Padalko A , Nair G, Sousa FL. Fusion/fission protein family identification in archaea. MSystems 2024;9:e0094823. 10.1128/msystems.00948-23 38700364 PMC11237513

[vbag069-B109] Parks DH , Chaumeil P-A, Mussig AJ et al GTDB release 10: a complete and systematic taxonomy for 715 230 bacterial and 17 245 archaeal genomes. Nucleic Acids Res 2026;54:D743–54. 10.1093/nar/gkaf1040 41123020 PMC12807784

[vbag069-B110] Parks DH , Chuvochina M, Rinke C et al GTDB: an ongoing census of bacterial and archaeal diversity through a phylogenetically consistent, rank normalized and complete genome-based taxonomy. Nucleic Acids Res 2022;50:D785–794. 10.1093/nar/gkab776 34520557 PMC8728215

[vbag069-B111] Parks DH , Imelfort M, Skennerton CT et al CheckM: assessing the quality of microbial genomes recovered from isolates, single cells, and metagenomes. Genome Res 2015;25:1043–55. 10.1101/gr.186072.114 25977477 PMC4484387

[vbag069-B112] Parks DH , Rinke C, Chuvochina M et al Recovery of nearly 8,000 metagenome-assembled genomes substantially expands the tree of life. Nat Microbiol 2017;2:1533–42. 10.1038/s41564-017-0012-7 28894102

[vbag069-B113] Pizza M , Scarlato V, Masignani V et al Identification of vaccine candidates against serogroup B meningococcus by Whole-Genome sequencing. Science 2000;287:1816–20. 10.1126/science.287.5459.1816 10710308

[vbag069-B114] Porras O , Caugant DA, Gray B et al Difference in structure between type b and nontypable Haemophilus influenzae populations. Infect Immun 1986a;53:79–89. 10.1128/iai.53.1.79-89.1986 3487508 PMC260078

[vbag069-B115] Porras O , Caugant DA, Lagergård T et al Application of multilocus enzyme gel electrophoresis to *Haemophilus influenzae*. Infect Immun 1986b;53:71–8. 10.1128/iai.53.1.71-78.1986 3522433 PMC260077

[vbag069-B116] Pritchard L , Glover RH, Humphris S et al Genomics and taxonomy in diagnostics for food security: soft-rotting enterobacterial plant pathogens. Anal Methods 2016;8:12–24. 10.1039/C5AY02550H

[vbag069-B117] Prondzinsky P , Toyoda S, McGlynn SE. The methanogen core and pangenome: conservation and variability across biology’s growth temperature extremes. DNA Res 2023;30:dsac048. 10.1093/dnares/dsac048 36454681 PMC9886072

[vbag069-B118] Puigbò P , Lobkovsky AE, Kristensen DM et al Genomes in turmoil: quantification of genome dynamics in prokaryote supergenomes. BMC Biol 2014;12:66. 10.1186/s12915-014-0066-4 25141959 PMC4166000

[vbag069-B119] Rafiq M , Hassan N, Rehman M et al Challenges and approaches of culturing the unculturable archaea. Biology (Basel) 2023;12:1499. 10.3390/biology12121499 38132325 PMC10740628

[vbag069-B120] Rajput A , Chauhan SM, Mohite OS et al Pangenome analysis reveals the genetic basis for taxonomic classification of the lactobacillaceae family. Food Microbiol 2023;115:104334. 10.1016/j.fm.2023.104334 37567624

[vbag069-B121] Rees CA , Nasir M, Smolinska A et al Detection of high-risk carbapenem-resistant *Klebsiella pneumoniae* and *Enterobacter cloacae* isolates using volatile molecular profiles. Sci Rep 2018;8:13297. 10.1038/s41598-018-31543-x 30185884 PMC6125577

[vbag069-B290] Reno ML , Held NL, Fields CJ et al Biogeography of the Sulfolobus islandicus pan-genome. Proc Natl Acad Sci 2009;106:8605–10. 10.1073/pnas.0808945106 19435847 PMC2689034

[vbag069-B122] Rice ES , Alberdi A, Alfieri J et al A pangenome graph reference of 30 chicken genomes allows genotyping of large and complex structural variants. BMC Biol 2023;21:267. 10.1186/s12915-023-01758-0 37993882 PMC10664547

[vbag069-B123] Rinke C , Schwientek P, Sczyrba A et al Insights into the phylogeny and coding potential of microbial dark matter. Nature 2013;499:431–7. 10.1038/nature12352 23851394

[vbag069-B124] Roder T , Pimentel G, Fuchsmann P et al Scoary2: rapid association of phenotypic multi-omics data with microbial pan-genomes. Genome Biol 2024;25:93. 10.1186/s13059-024-03233-7 38605417 PMC11007987

[vbag069-B125] Rodriguez-R LM , Konstantinidis KT. Bypassing cultivation to identify bacterial species. Microbe Magazine 2014;9:111–8. 10.1128/microbe.9.111.1

[vbag069-B126] Rouli L , Mbengue M, Robert C et al Genomic analysis of three African strains of *Bacillus anthracis* demonstrates that they are part of the clonal expansion of an exclusively pathogenic bacterium. New Microbes New Infect 2014;2:161–9. 10.1002/nmi2.62 25566394 PMC4265047

[vbag069-B127] Rouli L , Merhej V, Fournier P-E et al The bacterial pangenome as a new tool for analysing pathogenic bacteria. New Microbes New Infect 2015;7:72–85. 10.1016/j.nmni.2015.06.005 26442149 PMC4552756

[vbag069-B128] Sarton-Lohéac G , Romashchenko N, Train CM et al Reconstructing evolutionary histories with hierarchical orthologous groups. J Mol Evol 2025;93:740–64. 10.1007/s00239-025-10277-1 41269329 PMC12756263

[vbag069-B129] Schoch CL , Ciufo S, Domrachev M et al NCBI taxonomy: a comprehensive update on curation, resources and tools. Database (Oxford) 2020;2020:baaa062. 10.1093/database/baaa062 32761142 PMC7408187

[vbag069-B130] Selander RK , Levin BR. Genetic diversity and structure in *Escherichia coli* populations. Science 1980;210:545–7. 10.1126/science.6999623 6999623

[vbag069-B131] Setubal JC. Metagenome-assembled genomes: concepts, analogies, and challenges. Biophys Rev 2021;13:905–9. 10.1007/s12551-021-00865-y 35059016 PMC8724365

[vbag069-B132] Shapiro BJ. The population genetics of pangenomes. Nat Microbiol 2017;2:1574–1574. 10.1038/s41564-017-0066-6 29176697

[vbag069-B133] Shendure J , Ji H. Next-generation DNA sequencing. Nat Biotechnol 2008;26:1135–45. 10.1038/nbt1486 18846087

[vbag069-B134] Shovon MHJ , Imtiaz M, Biswas P et al A pan-genomic analysis based multi-epitope vaccine development by targeting *Stenotrophomonas maltophilia* using reverse vaccinology method: an in-silico approach. In Silico Pharmacol 2024;12:93. 10.1007/s40203-024-00271-8 39464855 PMC11499521

[vbag069-B135] Simão FA , Waterhouse RM, Ioannidis P et al BUSCO: assessing genome assembly and annotation completeness with single-copy orthologs. Bioinformatics 2015;31:3210–2. 10.1093/bioinformatics/btv351 26059717

[vbag069-B136] Simon SA , Aschmann V, Behrendt A et al Earth’s most needed uncultivated aquatic prokaryotes. Water Res 2025;273:122928. 10.1016/j.watres.2024.122928 39724798

[vbag069-B137] Smillie CS , Smith MB, Friedman J et al Ecology drives a global network of gene exchange connecting the human microbiome. Nature 2011;480:241–4. 10.1038/nature10571 22037308

[vbag069-B138] Soares SC , Silva A, Trost E et al The pan-genome of the animal pathogen *Corynebacterium pseudotuberculosis* reveals differences in genome plasticity between the biovar ovis and equi strains. PLoS One 2013;8:e53818. 10.1371/journal.pone.0053818 23342011 PMC3544762

[vbag069-B139] Springer E , Sachs MS, Woese CR et al Partial gene sequences for the a subunit of methyl-coenzyme M reductase (mcrI) as a phylogenetic tool for the family methanosarcinaceae. Int J Syst Bacteriol 1995;45:554–9. 10.1099/00207713-45-3-554 8590683

[vbag069-B140] Steinke K , Mohite OS, Weber T et al Phylogenetic distribution of secondary metabolites in the *Bacillus subtilis* species complex. MSystems 2021;6 10.1128/msystems.00057-21 PMC8546965 33688015

[vbag069-B141] Stewart FM , Levin BR. The population biology of bacterial plasmids: *a priori* conditions for the existence of conjugationally transmitted factors. Genetics 1977;87:209–28. 10.1093/genetics/87.2.209 17248761 PMC1213735

[vbag069-B142] Su P , Wicaksono WA, Li C et al Recovery of metagenome-assembled genomes from the phyllosphere of 110 rice genotypes. Sci Data 2022;9:254. 10.1038/s41597-022-01320-7 35650240 PMC9160027

[vbag069-B143] Tantoso E , Eisenhaber B, Kirsch M et al To kill or to be killed: pangenome analysis of *Escherichia coli* strains reveals a tailocin specific for pandemic ST131. BMC Biol 2022;20:146. 10.1186/s12915-022-01347-7 35710371 PMC9205054

[vbag069-B144] Taylor DJ , Eizenga JM, Li Q et al Beyond the human genome project: the age of complete human genome sequences and pangenome references. Annu Rev Genomics Hum Genet 2024;25:77–104. 10.1146/annurev-genom-021623-081639 38663087 PMC11451085

[vbag069-B145] Tettelin H , Masignani V, Cieslewicz MJ et al Genome analysis of multiple pathogenic isolates of *Streptococcus agalactiae*: implications for the microbial “pan-genome”. Proc Natl Acad Sci USA 2005;102:13950–5. 10.1073/pnas.0506758102 16172379 PMC1216834

[vbag069-B146] Tettelin H , Masignani V, Cieslewicz MJ et al Complete genome sequence and comparative genomic analysis of an emerging human pathogen, serotype V *Streptococcus agalactiae*. Proc Natl Acad Sci USA 2002;99:12391–6. 10.1073/pnas.182380799 12200547 PMC129455

[vbag069-B147] Tettelin H , Medini D. The Pangenome. Cham, Switzerland: Springer International Publishing, 2020. 10.1007/978-3-030-38281-0

[vbag069-B148] Tettelin H , Riley D, Cattuto C et al Comparative genomics: the bacterial pan-genome. Curr Opin Microbiol 2008;11:472–7. 10.1016/j.mib.2008.09.006 19086349

[vbag069-B149] Tettelin H , Saunders NJ, Heidelberg J et al Complete genome sequence of *Neisseria meningitidis* serogroup B strain MC58. Science 2000;287:1809–15. 10.1126/science.287.5459.1809 10710307

[vbag069-B150] Tong C , Jia Y, Hu H et al Pangenome and pantranscriptome as the new reference for gene-family characterization: a case study of basic helix-loop-helix (bHLH) genes in barley. Plant Commun 2025;6:101190. 10.1016/j.xplc.2024.101190 39521956 PMC11783906

[vbag069-B151] Tonkin-Hill G , Corander J, Parkhill J. Challenges in prokaryote pangenomics. Microb Genom 2023;9:mgen001021. 10.1099/mgen.0.001021 37227251 PMC10272878

[vbag069-B152] Tonkin-Hill G , MacAlasdair N, Ruis C et al Producing polished prokaryotic pangenomes with the panaroo pipeline. Genome Biol 2020;21:180. 10.1186/s13059-020-02090-4 32698896 PMC7376924

[vbag069-B153] Torres MDT , Wan F, de la Fuente-Nunez C. Deep learning reveals antibiotics in the archaeal proteome. Nat Microbiol 2025;10:2153–67. 10.1038/s41564-025-02061-0 40796684 PMC12408343

[vbag069-B154] Tyson GW , Banfield JF. Cultivating the uncultivated: a community genomics perspective. Trends Microbiol 2005;13:411–5. 10.1016/j.tim.2005.07.003 16043355

[vbag069-B155] Tyson GW , Chapman J, Hugenholtz P et al Community structure and metabolism through reconstruction of microbial genomes from the environment. Nature 2004;428:37–43. 10.1038/nature02340 14961025

[vbag069-B156] Vanin EF. Processed pseudogenes: characteristics and evolution. Annu Rev Genet 1985;19:253–72. 10.1146/annurev.ge.19.120185.001345 3909943

[vbag069-B210] Verma DK , Chaudhary C, Singh L et al Isolation and taxonomic characterization of novel Haloarchaeal isolates from Indian solar saltern: a brief review on distribution of bacteriorhodopsins and V-type ATPases in Haloarchaea. Front Microbiol 2020;11:1–14. 10.3389/fmicb.2020.554927 33362726 PMC7755889

[vbag069-B220] Wang P , Li LZ, Qin YL et al Comparative genomic analysis reveals the metabolism and evolution of the thermophilic archaeal genus metallosphaera. Front Microbiol 2020;11. 10.3389/fmicb.2020.01192 PMC7325606 32655516

[vbag069-B157] Wang J , Yang W, Zhang S et al A pangenome analysis pipeline provides insights into functional gene identification in rice. Genome Biol 2023;24:19. 10.1186/s13059-023-02861-9 36703158 PMC9878884

[vbag069-B950] Wang S , Narsing Rao MP, Quadri SR et al Assessing the metabolism, phylogenomic, and taxonomic classification of the halophilic genus Halarchaeum. FEMS Microbiol Lett 2024;371. 10.1093/femsle/fnae001 38192037

[vbag069-B158] Welch RA , Burland V, Plunkett G et al Extensive mosaic structure revealed by the complete genome sequence of uropathogenic *Escherichia coli*. Proc Natl Acad Sci USA 2002;99:17020–4. 10.1073/pnas.252529799 12471157 PMC139262

[vbag069-B159] Whitman WB , Coleman DC, Wiebe WJ. Prokaryotes: the unseen majority. Proc Natl Acad Sci USA 1998;95:6578–83. 10.1073/pnas.95.12.6578 9618454 PMC33863

[vbag069-B160] Woese CR , Fox GE. Phylogenetic structure of the prokaryotic domain: the primary kingdoms. Proc Natl Acad Sci USA 1977;74:5088–90. 10.1073/pnas.74.11.5088 270744 PMC432104

[vbag069-B161] Wu H , Wang D, Gao F. Toward a high-quality pan-genome landscape of *Bacillus subtilis* by removal of confounding strains. Brief Bioinform 2021;22:1951–71. 10.1093/bib/bbaa013 32065216

[vbag069-B162] Wu H , Yang Z-K, Yang T et al An effective preprocessing method for high-quality pan-genome analysis of *Bacillus Subtilis* and *Escherichia coli*. In: *microbial Pangenomes*. New York, NY: Humana Press, 2022, 371–90. 10.1007/978-1-0716-1720-5_21 34709628

[vbag069-B163] Yang T , Gao F. High-quality pan-genome of *Escherichia coli* generated by excluding confounding and highly similar strains reveals an association between unique gene clusters and genomic islands. Brief Bioinform 2022;23:bbac283. 10.1093/bib/bbac283 35809555

[vbag069-B164] Yang Z , Guarracino A, Biggs PJ et al Pangenome graphs in infectious disease: a comprehensive genetic variation analysis of *Neisseria meningitidis* leveraging oxford nanopore long reads. Front Genet 2023;14:1225248. 10.3389/fgene.2023.1225248 37636268 PMC10448961

[vbag069-B165] Yin Z , Liu X, Qian C et al Pan-Genome analysis of *Delftia tsuruhatensis* reveals important traits concerning the genetic diversity, pathogenicity, and biotechnological properties of the species. Microbiol Spectr 2022;10:e0207221. 10.1128/spectrum.02072-21 35230132 PMC9045143

[vbag069-B953] Yu Z , Ma Y, Zhong W et al Comparative genomics of Methanopyrus sp. SNP6 and KOL6 revealing genomic regions of plasticity implicated in extremely thermophilic profiles. Front Microbiol 2017;8. 10.3389/fmicb.2017.01278 PMC5504354 28744269

[vbag069-B166] Zeng L , Wang D, Hu N et al A novel pan-genome reverse vaccinology approach employing a negative-selection strategy for screening surface-exposed antigens against leptospirosis. Front Microbiol 2017;8:396. 10.3389/fmicb.2017.00396 28352257 PMC5348505

[vbag069-B167] Zha Y , Chong H, Yang P et al Microbial dark matter: from discovery to applications. Genomics Proteomics Bioinformatics 2022;20:867–81. 10.1016/j.gpb.2022.02.007 35477055 PMC10025686

[vbag069-B168] Zhang L , Kulyar MF, Niu T et al Comparative genomics of *Limosilactobacillus reuteri* YLR001 reveals genetic diversity and probiotic properties. Microorganisms 2024;12:1636. 10.3390/microorganisms12081636 39203478 PMC11356486

[vbag069-B169] Zhang Y , Jing H. Metagenome sequencing and 107 microbial genomes from seamount sediments along the Yap and mariana trenches. Sci Data 2024;11:887. 10.1038/s41597-024-03762-7 39147792 PMC11327340

[vbag069-B170] Zhang Y , Li R, Zou G et al Discovery of antimicrobial lysins from the “dark matter” of uncharacterized phages using artificial intelligence. Adv Sci (Weinh) 2024;11:e2404049. 10.1002/advs.202404049 38899839 PMC11348152

[vbag069-B952] Zhong C , Wang L, Ning K. Pan‐genome study of Thermococcales reveals extensive genetic diversity and genetic evidence of thermophilic adaption. Environ Microbiol 2021;23:3599–613. 10.1111/1462-2920.15234 32939951

[vbag069-B171] Zhou Y , Yang L, Han X et al Assembly of a pangenome for global cattle reveals missing sequences and novel structural variations, providing new insights into their diversity and evolutionary history. Genome Res 2022;32:1585–601. 10.1101/gr.276550.122 35977842 PMC9435747

[vbag069-B172] Zorigt T , Furuta Y, Paudel A et al Pan-genome analysis reveals novel chromosomal markers for multiplex PCR-based specific detection of *Bacillus anthracis*. BMC Infect Dis 2024;24:942. 10.1186/s12879-024-09817-9 39251928 PMC11385494

[vbag069-B173] Zwick ME , Joseph SJ, Didelot X et al Genomic characterization of the *Bacillus cereus* sensu lato species: backdrop to the evolution of *Bacillus anthracis*. Genome Res 2012;22:1512–24. 10.1101/gr.134437.111 22645259 PMC3409264

